# Radiosensitization by Kinase Inhibition Revealed by Phosphoproteomic Analysis of Pancreatic Cancer Cells

**DOI:** 10.1074/mcp.RA120.002046

**Published:** 2020-11-25

**Authors:** Svenja Wiechmann, Elena Saupp, Daniela Schilling, Stephanie Heinzlmeir, Günter Schneider, Roland M. Schmid, Stephanie E. Combs, Bernhard Kuster, Sophie Dobiasch

**Affiliations:** 1 Chair of Proteomics and Bioanalytics, Technical University of Munich, Freising, Germany; 2 German Cancer Consortium, Munich, Germany; 3 German Cancer Center, Heidelberg, Germany; 4 Department of Radiation Oncology, Technical University of Munich, Klinikum rechts der Isar, Munich, Germany; 5 Institute of Radiation Medicine, Department of Radiation Sciences, Helmholtz Zentrum München, Neuherberg, Germany; 6 Medical Clinic and Polyclinic II, Klinikum rechts der Isar, Technical University Munich, München, Germany; 7 Bavarian Center for Biomolecular Mass Spectrometry, Technical University of Munich, Freising, Germany

**Keywords:** Pancreatic cancer, cancer therapeutics, phosphoproteome, kinases, enzyme inhibition, kinase inhibitors, kinase substrates, radioresistance

## Abstract

Pancreatic ductal adenocarcinoma (PDAC) is one of the most aggressive cancers and known for its extensive genetic heterogeneity, high therapeutic resistance, and strong variation in intrinsic radiosensitivity. To understand the molecular mechanisms underlying radioresistance, we screened the phenotypic response of 38 PDAC cell lines to ionizing radiation. Subsequent phosphoproteomic analysis of two representative sensitive and resistant lines led to the reproducible identification of 7,800 proteins and 13,000 phosphorylation sites (p-sites). Approximately 700 p-sites on 400 proteins showed abundance changes after radiation in all cell lines regardless of their phenotypic sensitivity. Apart from recapitulating known radiation response phosphorylation markers such as on proteins involved in DNA damage repair, the analysis uncovered many novel members of a radiation-responsive signaling network that was apparent only at the level of protein phosphorylation. These regulated p-sites were enriched in potential ATM substrates and *in vitro* kinase assays corroborated 10 of these. Comparing the proteomes and phosphoproteomes of radiosensitive and -resistant cells pointed to additional tractable radioresistance mechanisms involving apoptotic proteins. For instance, elevated NADPH quinine oxidoreductase 1 (NQO1) expression in radioresistant cells may aid in clearing harmful reactive oxygen species. Resistant cells also showed elevated phosphorylation levels of proteins involved in cytoskeleton organization including actin dynamics and focal adhesion kinase (FAK) activity and one resistant cell line showed a strong migration phenotype. Pharmacological inhibition of the kinases FAK by Defactinib and of CHEK1 by Rabusertib showed a statistically significant sensitization to radiation in radioresistant PDAC cells. Together, the presented data map a comprehensive molecular network of radiation-induced signaling, improves the understanding of radioresistance and provides avenues for developing radiotherapeutic strategies.

## Teaser

The proteomes and phosphoproteomes of radiosensitive and radioresistant PDAC cell lines were analyzed. Irrespective of the sensitivity of the cells, the phosphorylation-based radiation-responsive signaling network featured known DDR proteins and novel ATM substrates. Radioresistant cells displayed significant expression levels of apoptotic proteins, including NQO1, and elevated phosphorylation levels of proteins involved in actin dynamics and FAK activity. Sensitization of former resistant PDAC cells toward radiation was realized by pharmacological inhibition of FAK and CHEK by Defactinib and Rabusertib.

## Graphical Abstract

**Figure:**
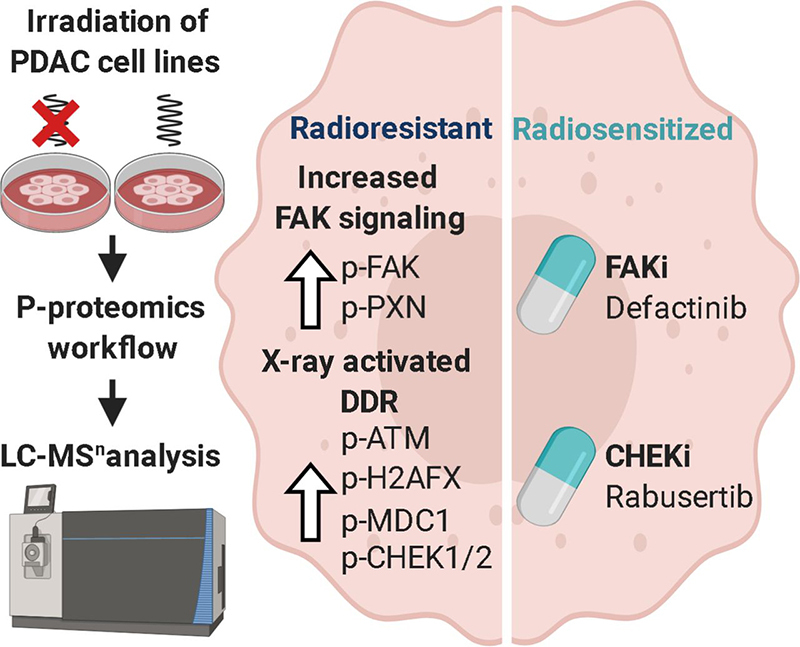

**Highlights**

• Proteomes and phosphoproteomes of radiosensitive and radioresistant PDAC cell lines.
• Common activation of DDR is proven by ATM activity on known and novel substrates.
• Resistant cells bear raised NQO1 expression, actin dynamics including FAK activity.
• Inhibitors of CHEK Rabusertib and FAK Defactinib radiosensitize PDAC cells.

Pancreatic ductal adenocarcinoma (PDAC) is one of the most aggressive and lethal cancers. Despite intense efforts in research and clinical care, the overall 5-year survival rate of patients with PDAC has only modestly improved in recent years and remains below 10% (1). Moreover, PDAC is projected to become the second leading cause of cancer-related deaths in the United States by 2030 (2). Among other factors, the poor survival of PDAC patients can be attributed to the generally late detection of the disease, high genetic heterogeneity, and strong therapeutic resistance. Therefore, pancreatic cancer continues to be challenging to treat and developing early detection as well as effective treatment regimens is an urgent need (3). Previous clinical trials have shown efficacy of radiation therapy (RT) in a subgroup of PDAC patients, and a positive impact of RT in the multimodal treatment of PDAC patients was observed. Patients with primary nonresectable, locally advanced PDAC can undergo neoadjuvant combined chemoradiation or RT for tumor downsizing with the aim of enabling a secondary resection and, therefore, achieving an improved long-term prognosis (4). In addition, a phase 2 study employing a neoadjuvant approach including systemic chemotherapy followed by individualized chemoradiotherapy for the treatment of borderline pancreatic cancer resulted in high rates of R0 resections and prolonged survival (5). The LAP07 phase 3 randomized trial showed a significantly decreased local progression and low toxicity rates in patients with locally advanced PDAC treated with chemoradiotherapy after induction chemotherapy when compared with chemotherapy alone (5). Still, the role of RT as a treatment option for PDAC patients is controversially discussed in the literature (6). Standardized guidelines are lacking as RT fails in about 70% of all cases because of the high genetic diversity and the heterogeneous intrinsic radiosensitivity of tumors, the presence of pancreatic cancer stem cells, the tumor microenvironment and nonconsideration of molecular profiles for therapy decisions (7, 8). Therefore, there is a clear clinical need to better characterize the radiation response of PDAC in order to be able to select the approximately 30% of patients who benefit from RT and to further increase the efficacy of RT (9).

Gamma radiation produces DNA lesions, particularly double strand breaks (DSB). Irradiated cells subsequently initiate a molecular process termed DNA damage response (DDR), which is tightly regulated by the activity of several kinases feeding into signaling cascades. Failure to repair DNA efficiently leads to cell death, the desired outcome of radiation therapy. Mechanisms by which tumors evade the lethal effect of radiation are manifold. One molecular hallmark of PDAC is desmoplasia, which is characterized by a dense extracellular matrix and may contribute to both radiation- and chemoresistance (10, 11). Hypoxia is another feature of PDAC (12), which may contribute to the low response to RT (13). In addition, the onset and progression of PDAC is driven by a large number of genetic mutations including alterations in the KRAS, TP53, SMAD4, and CDKN2A genes that often activate oncogenic or inhibit tumor suppressing signaling pathways and also impact the response to radiation (14).

As DNA damage repair and the aforementioned pathways are critically controlled in their activities by kinases and phosphatases, a better understanding of the similarities and differences in the phosphoproteomes of radiosensitive and radioresistant PDAC cells may identify opportunities to sensitize PDACs to RT. Toward this goal, we screened a panel of 38 PDAC cell lines for response to radiation and analyzed resistant and sensitive models by a combined proteomic and phosphoproteomic approach. These data constitute a comprehensive map of radiation induced signaling, identified novel substrates for the kinase ATM and defined Rabusertib and Defactinib, inhibitors of CHEK1 and FAK respectively, as radiosensitizers.

## EXPERIMENTAL PROCEDURES

##### Experimental Design and Statistical Rationale

The overall experimental design comprised an initial screening step to determine the radiation response of 38 PDAC cell lines. From the screen, two representative sensitive and two representative resistant cell lines were chosen as examples for subsequent proteomic and phosphoproteomic characterization as well as for further follow-up experiments targeting radioresistance. All experiments were replicated and the number of replicates performed for each type of experiment is provided in the respective sections.

All cell line *in vitro* radiation and inhibitor treatment data of all independent experiments were calculated as mean ± S.D. Statistical analysis was performed using GraphPad Prism software (version 8.0.2, GraphPad software Inc., San Diego, CA). Statistical significance between treated groups (kinase inhibitor and or irradiation) and an untreated control group was determined by multiple t-tests using the Holm-Sidak method. An adjusted p-value of < 0.05 was statistically significant (*p ≤ 0.05, **p ≤ 0.01, ***p ≤ 0.001, ****p ≤ 0.0001).

##### Cell Lines and Cell Culture

In this study, we took advantage of the availability of a previously described primary murine PDAC cell line panel (15). Briefly, these cell lines were derived from genetically engineered Ptf1a/p48ex1Cre/+;LSL-KRASG12D/+ or Ptf1a/p48ex1Cre/+;LSL-KRASG12D/+;TP53lox/lox mice. From this panel, 38 low-passaged lines with various genetic backgrounds were selected for this study (supplemental Table S1A). Cells were cultured in Dulbecco's Modified Eagle's Medium - high glucose medium (Sigma-Aldrich, St. Louis, MO) supplemented with 10% fetal calf serum (FCS), 100 U/ml penicillin and 100 μg/ml streptomycin (Invitrogen GmbH, Karlsruhe, Germany) at 37 °C in a humidified 5% CO_2_ atmosphere. Cells were routinely checked for mycoplasma contamination using the MycoAlert^TM^ Mycoplasma Detection Kit (Lonza Group, Basel, Switzerland).

##### Irradiation Experiments

Radiation was delivered at 220 kV and 15 mA with a dose rate of 0.90 Gy/min using the RS225A irradiation device (Gulmay/Xstrahl, Camberley, UK).

##### Screening for Radioresistance

The aforementioned 38 PDAC cell lines were screened for radiation response using the AlamarBlue proliferation assay. Cells were either irradiated with 0 Gy, 2 Gy, 4 Gy, 8 Gy or 16 Gy 24 h after seeding. The AlamarBlue reagent (Thermo Fisher Scientific, Waltham, MA) was added 72 h after irradiation. After an incubation time of four hours at 37 °C in a humidified 5% CO_2_ atmosphere, proliferation of cells was measured by absorbance at 570 nm and 630 nm using a microplate reader (ELx808™, BioTek, Winooski, VT). Data were captured by the Gen5 Software (BioTek, Winooski, VT) and analyzed in Microsoft Excel (supplemental Table S1A). The proliferation rate after irradiation with 8 Gy from at least two independent experiments was used to evaluate the response to radiation. Two representative cell lines with a high (53704PPT, F5461PPT2) and low (53578PPT, 5748PPT) radioresponse were chosen for further analyses.

##### Clonogenic Survival after Radiation Treatment

Colony formation assays (CFA) were performed to determine the radiosensitivity and confirm the data from the AlamarBlue proliferation screening assay. Cells were plated into 12-well plates and irradiated with different doses (0 Gy, 2 Gy, 4 Gy, 6 Gy, and 8 Gy) 48 h after seeding. Seven to 9 days (depending on the cell line) after plating, the colonies were fixed with ice-cold methanol, stained with 0.1% crystal violet and counted with the GelCount™ apparatus (Oxford Optronics, Abingdon, UK). Colonies of more than 50 single cells were defined as one colony. Survival curves were fitted according to the following linear-quadratic model with the equation “ln SF = −α × D − β × D2” by the GraphPad Prism Software. Each experiment was at least performed thrice (supplemental Table S1B).

##### Migration Assay

To determine the migration behavior of the two representative radioresistant and radiosensitive cell lines, Corning Control Chambers were used according to manufacturer's instructions. Control inserts for the investigation of migration contain an uncoated 8 μm pore size membrane (24-well, Catalog #354578, Corning, New York). Medium containing 10% FCS was used as an attractant in the lower chamber. 10 × 10^3^ cells in medium containing 0.5% FCS (serum starvation) per insert were seeded in the upper compartment of the insert and were incubated for 20 h in a humidified incubator at 37 °C and 5% CO_2_ atmosphere. Nonmigrated cells were then removed from the upper surface of the membrane and migrated cells were fixed with ice-cold methanol and stained with 0.1% crystal violet. Cells from five independent fields of each membrane were counted using an AxioImager Z1 microscope (Zeiss, Oberkochen, Germany) at 10 × magnification. All invasion assays were performed at least in duplicate in at least 5 independent experiments. Migration was quantified by calculating the mean number of cells migrating through the uncoated membrane. Statistical significance between the migration capacities of the different cell lines was determined by unpaired t-tests (supplemental Table S6C).

##### Sample Preparation for (Phospho)Proteomic Experiments in Response to Radiation

F5461PPT2, 53704PPT, 53578PPT and 5748PPT cells were seeded and 24 h later irradiated with either 0 Gy or 8 Gy. Each experiment was performed three times.1 h after irradiation, cells were washed twice with PBS and lysed in 40 mm Tris-HCl pH 7.6, 8 M urea, EDTA-free protease inhibitor (Roche) and phosphatase inhibitors (Roche). Lysates were centrifuged for 1 h at 21,000 × *g* und the supernatant was subjected to sample preparation. The protein concentration in cell lysate was determined using the Coomassie Plus Bradford (Thermo Fisher Scientific) assay according to the protocol of the manufacturer. Lysate of either 0 Gy (mix1) or 8 Gy (mix2) treated cells was in part pooled in a 1:1:1:1 ratio and processed along with the individual lysates.

Two hundred micrograms protein of cell lysate was reduced with 10 mm DTT for 40 min at 56 °C and alkylated with 55 mm chloroacetamide (CAA) at room temperature in the dark for 20 min. After dilution of the urea concentration from 6 M to 1.5 M with 40 mm Tris-HCl pH 7.6, the proteins were digested in a 1:50 trypsin/substrate weight ratio overnight at 37 °C and 700 rpm. Desalting of the tryptic peptides was performed on Sep-Pak C18 50 mg columns (Waters) as described elsewhere in 0.07% TFA in 50% acetonitrile (ACN). Afterward labeling of the desalted peptides was performed with tandem mass tags 10 (TMT10)-plex (Thermo Fisher Scientific) at a final concentration of 6.67 mm TMT according to instructions provided by the manufacturer except for a 1:2 TMT/peptide ratio. One TMT channel was used for each cell line and treatment condition (126 = 53578PPT-0 Gy, 127N = 53578PPT-8 Gy, 127C = 53704PPT-0 Gy, 128N = 53704PPT-8 Gy, 128C = F5461-0 Gy, 129N = F5461-8 Gy, 129C = 5748PPT-0 Gy, 130N = 5748PPT-8 Gy, 13 °C = mix-0 Gy, 131 = mix-8 Gy). Pooled phosphopeptides were enriched using Fe-immobilized metal ion affinity chromatography (IMAC) as previously described (16). Subsequently, phosphopeptides were separated into six fractions using basic reversed-phase (bRP) chromatography in micro-column format (five disks, Ø 1.5 mm, C18 material, 3M Empore per micro-column were used) in 25 mm NH_4_COOH (pH 10). Peptides were fractionated with increasing ACN concentrations (5%, 7.5%, 10%, 12.5%, 15%, 17.5, and 50% ACN). The desalted flow-through was combined with the 17.5% fraction and the 50% fraction with the 5% fraction to give a total of six fractions. Nonphosphorylated peptides (flow through of the Fe-IMAC) were fractionated into 32 fractions by trimodal mixed mode chromatography, which uses reversed phase, weak anion exchange, and strong cation exchange, as published (17). After drying in a centrifugal evaporator, the samples were stored at −20 °C until LC-MSn analysis.

##### LC-MSn Analysis of the (Phospho)Proteome in Response to Radiation

Nano-flow LC-MSn measurement of TMT-labeled nonphosphorylated and phosphorylated peptides was performed using a Dionex Ultimate3000 nano HPLC (Thermo Fisher Scientific) coupled to an Orbitrap Fusion Lumos mass spectrometer (Thermo Fisher Scientific). The fractions of phosphorylated peptides were injected twice. Peptides were desalted on a trap column (100 μm × 2 cm, packed in-house with Reprosil-Pur C18-AQ 5 μm resin; Dr. Maisch) in 0.1% formic acid (FA) at 5 µl/min and separated on an analytical column (75 μm × 40 cm, packed in-house with Reprosil-Pur C18-AQ, 3 μm resin; Dr. Maisch) using a 50 min linear gradient from 8-34% (full proteome) or 80 min linear gradient from 4-32% (phosphoproteome) solvent B (0.1% FA, 5% DMSO in ACN) in solvent A (0. 1% FA, 5% DMSO in water) at a flow rate of 300 nL/min. The Fusion Lumos was operated in data dependent acquisition and positive ionization mode. Full scan MS1 spectra were acquired over a range of 360–1300 *m*/*z* at a resolution of 60,000 (automatic gain control (AGC) target value 4e5, maximal injection time 10 ms). For LC-MS2 analysis of the full proteome, up to 20 peptide precursors were selected for fragmentation by higher energy collision-induced dissociation (HCD; 1.2 *m*/*z* isolation window, AGC value of 2e5, maximum injection time of 50 ms) using 38% normalized collision energy (NCE) and analyzed at a resolution of 30,000 in the Orbitrap. For LC-MS3 analysis of the phosphoproteome, up to 10 peptide precursors were selected for fragmentation by collision-induced dissociation (CID; 0.7 *m*/*z* isolation window, AGC value of 5e4, maximum injection time of 60 ms) using 35% collision energy and analyzed at a resolution of 30,000 in the Orbitrap. An additional MS3 spectrum was acquired in the orbitrap over a m/z range of 100–1000 at 50,000 resolution for each peptide precursor. For this, fragment ions were selected by multi-notch isolation, allowing a maximum of 10 notches and an ion trap isolation width of 2 Da, and subsequently fragmented by HCD at 55% NCE (AGC target value 1.2e5, maximal injection time 120 ms).

##### Peptide and Protein Identification and Quantification

Protein and peptide identification and quantification was performed using MaxQuant (18) (version 1.5.6.5) by searching the tandem MS data against all mouse canonical sequences as annotated in the Swissprot reference database (16,889 entries, downloaded 27.06.2017) using the search engine Andromeda (19). Carbamidomethylated cysteine was set as fixed modification and oxidation of methionine and N-terminal protein acetylation as variable modification. In addition, phosphorylation of serine, threonine and tyrosine was set as variable modification for the phosphoproteome. Trypsin/P was specified as the proteolytic enzyme and up to two missed cleavage sites were allowed. Precursor tolerance was set to 4.5 ppm and fragment ion tolerance to 20 ppm. The minimum peptide length was set to seven and all data were adjusted to 1% peptide-spectrum match (PSM) and 1% protein false discovery rate (FDR). A minimum score for modified peptides was set to 40. MS2- (full proteome) and MS3-based (phosphoproteome) TMT quantification was enabled, taking TMT correction factors as supplied by the manufacturer into account. Subsequent data analysis was performed on identified and quantified protein groups (full proteome; as provided in the proteinGroups.txt; supplemental Table S2A) and phosphorylation sites (phosphoproteome; as provided in the Phospho (STY)Sites.txt; supplemental Table S2B).

##### In-Vitro ATM Kinase Assays

Synthetic peptides were designed as 15-mers (if not stated otherwise) with serine or alanine in the central position for WT or mutant p-sites, respectively, in two peptide pools (supplemental Table S4B). Peptides were supplied by JPT Peptide Technologies GmbH (Germany) in two pools. Each peptide pool was subjected to separate kinase assays. Peptides were added at a concentration of 3 μm (pool 1) and 4.6 μm (pool 2) to 50 mm HEPES-KOH pH 7.4, 150 mm NaCl, 6 mm MgCl_2_, 4 mm MnCl2, 1 mm DTT and 2 mm ATP. The assay was started by adding 300 ng recombinant active ATM (Sigma-Aldrich, #14-933-M; ATM samples) or vehicle (control samples) and the reaction could proceed at 30 °C for 1 h. Each kinase assay was performed in triplicate (if not stated otherwise). The reaction was quenched by adding an equal volume of 1% FA in ACN. After drying in a centrifugal evaporator, samples were stored at −20 °C until LC–MS/MS analysis.

##### Proteome Data Analysis

The Perseus software suite (20) (version 1.5.5.3) was used to filter out contaminants and reverse hits. For the full proteome data set, protein groups that were only identified by a modified peptide were also removed. Furthermore, only phosphorylation sites and protein groups that were detected in at least two out of the three replicates were considered for further analysis. Log2 fold changes for 8 Gy against 0 Gy control and resistance against sensitivity samples were calculated per phosphorylation site and protein group and tested for significance using a *t* test (FDR = 1% or 5%, s0 = 0.1). Protein-protein interactions were analyzed using the String database (21) (version 11.0) (combined score > 0.4) and visualized in Cytoscape (version 3.4.0). The PANTHER Classification System was used for gene ontology (GO) enrichment analysis (22) (supplemental Tables S3C, S5C). Identified and quantified p-sites of the synthetic peptides from the *in vitro* kinase assays were only considered as ATM substrates if they (1) were not detected in the control samples in more than one replicate and (2) not detected in the kinase reaction of the mutant peptide.

##### LC–MS/MS Analysis of the Kinase Assay

Nano-flow LC–MS/MS of peptides from the *in vitro* kinase assay was performed using a Dionex Ultimate3000 nano HPLC (Thermo Fisher Scientific) coupled to an Orbitrap Fusion Lumos mass spectrometer (Thermo Fisher Scientific). Peptides were desalted on a trap column (100 μm × 2 cm, packed in-house with Reprosil-Pur C18-AQ 5 μm resin; Dr. Maisch) in 0.1% FA and separated on an analytical column (75 μm × 40 cm, packed in-house with Reprosil-Pur C18-AQ, 3 μm resin; Dr. Maisch) using a 51 min two-step gradient from 4-15-27% B (0.1% FA, 5% DMSO in 100% ACN) in solution A (0.1% FA, 5% DMSO in water). The Fusion Lumos was operated in data dependent acquisition and positive ionization mode. Full scan MS1 spectra were acquired over a range of 360-1300 m/z at a resolution of 60,000 (AGC target value 4 × 10^5^, maximal injection time 50 ms). Fragmentation was performed using HCD at 30% NCE (AGC target value 5 × 10^4^, maximal injection time 120 ms) in the orbitrap at 30,000 resolution.

##### Peptide and Protein Identification and Quantification of the Kinase Assay

Peptide identification and quantification was performed by searching the MS data using MaxQuant (18) (version 1.6.0.1) against a database containing only sequences of the screened peptides. Phosphorylation of serine and threonine, oxidation of methionine, and N-terminal protein acetylation were set as variable modifications. Precursor and fragment ion tolerances were 4.5 ppm and 20 ppm, respectively. Subsequent data analysis was performed on identified and quantified protein groups phosphorylation sites (provided in the Phospho(STY)Sites.txt; supplemental Table S4D).

##### Western Blotting

Protein lysates were generated by harvesting cells in 0.8% Nonidet P-40, 50 mm Tris-HCl pH 7.5, 5% glycerol, 1.5 mm MgCl_2_, 150 mm NaCl, 1 mm Na_3_VO_4_, 25 mm NaF, 1 mm DTT, protease inhibitors (SigmaFast, Sigma) and phosphatase inhibitors. Proteins were separated by SDS-PAGE and electro-transferred onto PVDF membranes. Blots were stored in TBS (TBS), supplemented with 0.05% Tween (TBS-T) and 2% BSA (BSA) for 1 h at room temperature and then incubated with primary antibody (diluted in TBS-T, 5% BSA) overnight at 4 °C. The following primary antibodies were used: alpha-tubulin (1:1000, Santa Cruz Biotechnology, # sc-5286), Phospho-FAK (Tyr576/577) (1:500, Cell Signaling Technology, #3281), FAK (1:1000, Cell Signaling Technology, #3285). Subsequently, blots were washed in TBS-T and probed with the corresponding fluorophore-conjugated secondary antibody for 30 min at room temperature. The immunoreactive signals were detected directly by excitation of the respective fluorophore. Acquisition and quantification of the band intensities were carried out with the Odyssey (Licor) imaging system and ImageJ. Intensities of proteins were normalized to input housekeeping proteins and phosphorylated proteins were normalized to the intensity of the respective total protein.

##### Small Molecule Kinase Inhibitors

The potent and selective FAK inhibitor Defactinib (#S7654, Selleckchem, Houston, TX) as well as the potent and highly selective Chek-1 inhibitor Rabusertib (#S2626, Selleckchem, Houston, TX) were used to target radioresistance. The kinase selectivity data for these compounds are provided in (23) and ProteomicsDB (24, 25). Both inhibitors were dissolved in DMSO to prepare a stock solution of 5 mm. All inhibitor treatments resulted in a maximal final DMSO concentration of 0.5% in the cell culture medium to avoid poisoning the cells. Initially, PDAC cells were treated with Defactinib in a concentration range from 500 nm to 10 μm or with Rabusertib from to 125 nm to 1 μm for 24 h to identify appropriate inhibitor concentrations for further experiments. In addition, CFAs including a DMSO control (0.5% DMSO) and an untreated control with PBS (PBS) were performed. Clonogenic cell survival in response to the drugs, 0.5% DMSO or PBS was determined after 7 days (supplemental Table S7A). Each experiment was at least performed three times. The survival curves were fitted according to the equation “Dose-response - Inhibition; log[inhibitor] *versus* normalized response – variable slope” with the bottom fit constrained to values between 0 and 1 using the GraphPad Prism software. Corresponding EC50 values were determined by this method.

##### Clonogenic Survival after Combination Treatment

A CFA was performed to evaluate the clonogenic cell survival after a combination treatment of kinase inhibitors and irradiation. Cells were treated with concentrations of 0 μm, 1 μm, 2 μm and 5 μm Defactinib or 0 nm, 125 nm, 250 nm and 375 nm Rabusertib 24 h after seeding and irradiated with 0 Gy, 2 Gy, 4 Gy, 6 Gy and 8 Gy another 24 h later. Immediately after radiation treatment, Defactinib or Rabusertib was removed from cells by changing the culture medium. Colonies were allowed to grow under normal cell culture conditions for 5 days after completion of the combined treatment. Seven days after plating, the colonies were fixed with ice-cold methanol, stained with 0.1% crystal violet, counted with the GelCount™ and survival curves were fitted by the linear-quadratic model. Each experiment was at least performed three times.

All cell survival data post-treatment were normalized to the unirradiated control sample at the respective inhibitor concentration (supplemental Table S7B).

##### Radiobiological Analysis after Combination Treatment

Radiobiological parameters including D_50_ (dose [Gy] to reduce survival fraction to 50%), the sensitizing enhancement ratio (SER) as well as the α and β values were derived from the linear quadratic equation SF = exp [−α × D − β × D2] mentioned above and were determined to confirm the radiosensitizing effect of Defactinib and Rabusertib. The SER was calculated as the ratio between D_50_ (irradiation) and D_50_ (irradiation and inhibitor). A SER greater than 1.20 was defined to be indicative for radiosensitization. The results of the radiobiological analysis are summarized in supplemental Table S7C.

## RESULTS

##### Heterogeneous Radiosensitivity in Murine Cell Lines with Different Genetic Backgrounds

To identify molecular factors underlying radiosensitivity and -resistance in PDAC, an extensive primary murine PDAC cell line panel was used. Many of the molecular principles underlying PDAC evolution and phenotypic diversification have been shown to be represented within this panel. The panel also mimics several aspects of human PDAC, such as subtypes with KRAS amplification, different routes of tumor development, such as chromothripsis, and a wide spectrum of genetic, morphological, and clinical heterogeneity (26).

**Fig. 1:**
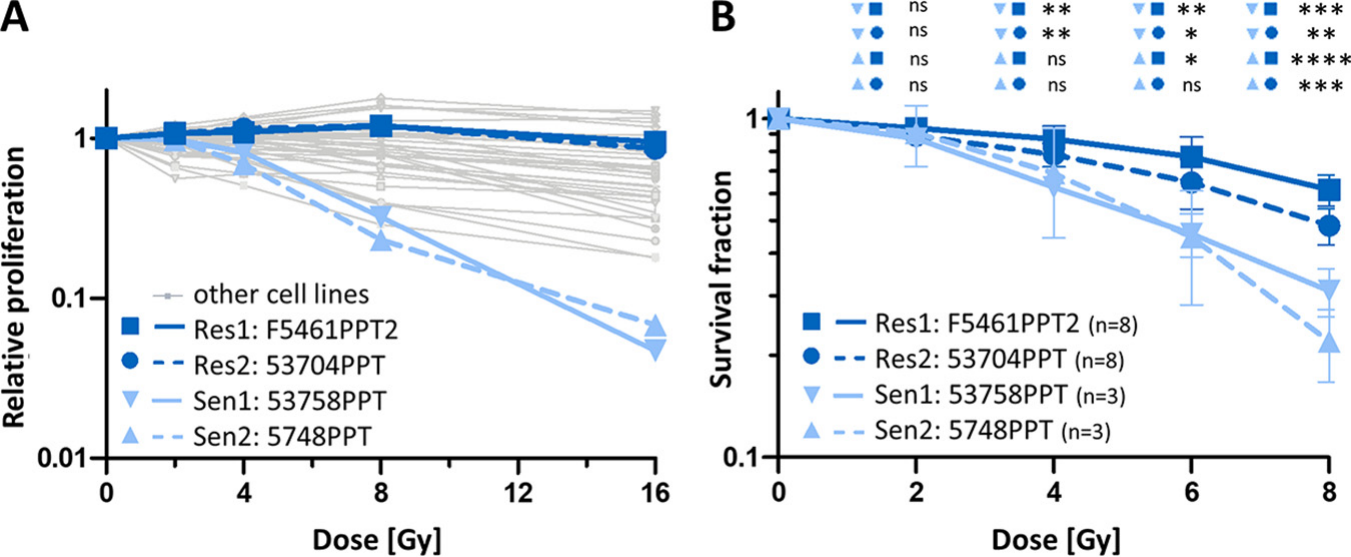
Radiation response of murine PDAC cell lines. *A*, Relative cell proliferation of 38 murine PDAC cell lines after irradiation with 0 Gy, 2 Gy, 4 Gy, 8 Gy and 16 Gy. Data are expressed as mean of at least 2 independent experiments. Two representative radioresistant (dark *blue*; F5461PPT2 (solid) and 53704PPT (dashed)) and two radiosensitive (light *blue*; 53578PPT (solid) and 5748PPT (dashed)) cell lines are highlighted. *B,* Clonogenic survival of the cell lines highlighted in panel A after irradiation with 0 Gy, 2 Gy, 4 Gy, 6 Gy and 8 Gy. Data are expressed as mean ± S.D. (*p ≤ 0.05, **p ≤ 0.01, ***p ≤ 0.001, ****p ≤ 0.0001).

First, we screened 38 murine PDAC cell lines with diverse genetic backgrounds (supplemental Table S1A) for their response to radiation using an AlamarBlue cell proliferation assay. The results show that the cell lines differ >10-fold in their radioresponse (Fig. 1*A*). Based on the median proliferation rate after irradiation with 8 Gy, we selected two radioresistant (F5461 PPT2, 53704 PPT) and two radiosensitive (53578 PPT, 5748 PPT) cell lines for further characterization and analysis. The radiation response data above were confirmed by colony formation assays (CFA) for these four cell lines (Fig. 1*B*, supplemental Table S1B). The fraction of surviving cells (survival fraction; SF) of the different PDAC cell lines statistically significantly differed at 4 Gy, 6 Gy, and 8 Gy radiation dose. The radioresistant cell line 53704 PPT showed a significantly higher SF compared with both radiosensitive cell lines. A significantly higher level of clonal survival after irradiation was observed for the radioresistant cell line F5461PPT2 compared with both radiosensitive cell lines 5748PPT and 53578 PPT. Consequently, the doses [Gy] required to reduce the survival fraction to 50% (D_50_) were significantly higher in the radioresistant 53704 PPT (7.89 Gy) and F5461 PPT2 (9.97 Gy) cells than in the radiosensitive cell lines 5748 PPT (5.48 Gy) and 53578 PPT (5.57 Gy). The results of the radiosensitivity as well as the radiobiological parameters are summarized in supplemental Table S1A–S1B.

##### Phosphoproteomic Analysis Reveals Common Radiation-Induced Signaling Changes in Sensitive and Resistant Cell Lines

**Fig. 2:**
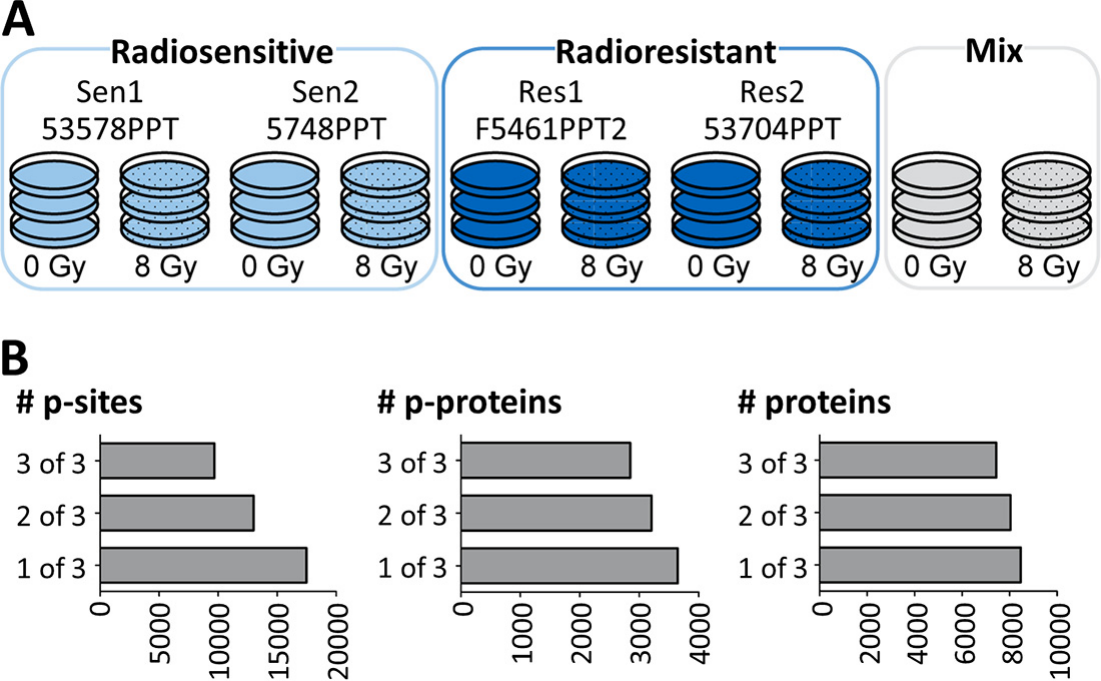
Proteomic and phosphoproteomic characterization of radiosensitive and radioresistant PDAC cell lines. *A*, Experimental setup using tandem mass tags (TMT-10plex). In each of three independent 10-plex TMT experiments, peptides from the four representative PDAC cell lines from Fig. 1. as well as a mixture of the same cell lines were included with and without radiation at 8 Gy. Radiosensitive cells are colored in light blue, radioresistant cells in dark blue. *B*, Summary of the number of proteins, phospho-proteins (p-proteins) and phosphorylation sites (p-sites) identified in one, two or three of three replicates.

To identify molecular factors underlying the observed radiation-induced phenotypic effects, the changes in the phosphoproteomes of two representative radiosensitive (53578 PPT and 5748 PPT) and two radioresistant (F5461 PPT2 and 53704 PPT) PDAC cell lines in response to radiation with X-rays (8 Gy; cells were collected 1 h after irradiation) were characterized. The use of tandem mass tags (TMT-10plex) (27) enabled the parallel analysis of the four cell lines and two treatment conditions along with a mixture of the untreated and treated cells in a single experiment (performed in triplicate; Fig. 2*A*; supplemental Fig. S1A). The analysis led to the identification of a total of ∼8,500 proteins, ∼3,600 phospho-proteins (p-proteins), ∼19,800 phosphopeptides (p-peptides) and ∼17,500 phosphorylation sites (p-sites; Fig. 2*B*; supplemental Tables S2A–S2C) in all ten samples making it the most comprehensive proteomic study of radiation-induced effects in cancer cells to date (28, 29, 30, 31, 32) (supplemental Fig. S1B). Replicates of proteomic experiments showed high correlation (median Pearson correlation coefficient for p-proteomes of *r* = 0.966 and for proteomes of *r* = 0.998) and low coefficients of variation between replicates (CV; median of 12% for p-sites and 2% for proteins) indicating generally high data consistency (supplemental Fig. S1C–S1D, supplemental Tables S2D–S2E). For all subsequent data analysis, only those p-sites and proteins were considered, which were identified in at least two of three replicates.

**Fig. 3:**
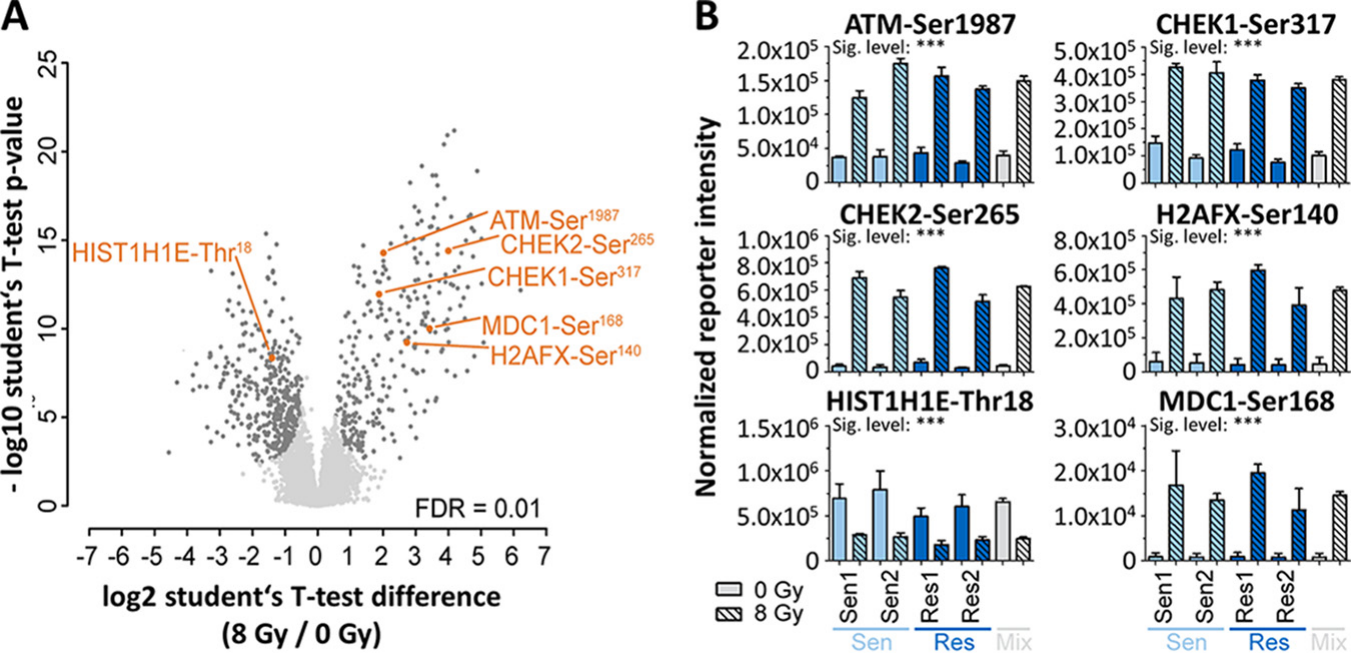
Phosphoproteomic response of PDAC cells to 8 Gy radiation. *A*, Volcano plot comparing the fold-change difference of irradiated and nonirradiated cells and the statistical significance of the observed differences. Individual p-sites of proteins of interest are highlighted in orange. *B*, Examples for p-site intensities on proteins of interest that show statistically significant differences between irradiated and nonirradiated cells.

A global comparison of irradiated *versus* nonirradiated cell lines (irrespective of their sensitivity to radiation) showed statistically significant quantitative changes of 747 p-sites on 409 proteins (Fig. 3*A*; supplemental Table S3A; 1% false discovery rate, FDR)), which took effect mostly irrespective of their sensitivity to radiation (supplemental Table S3B). These included several well-known DDR markers, such as increased phosphorylation at ATM-Ser^1987^ (an autophosphorylation event, which is suspected to increase ATM activity in response to DNA DSB (33, 34)), the direct ATM substrate H2AFX-Ser^140^ (35), the recruitment and phosphorylation of MDC1-Ser^168^ (reader protein of H2AFX-Ser^140^) (36), the indirect ATM substrate CHEK2-Ser^265^ and the ATR substrate CHEK1-Ser^317^ (37) (Fig. 3*B*). Conversely, phosphorylation at HIST1H1E-Thr^18^, a renowned ATM-dependent histone marker (38), was significantly decreased upon radiation. GO analysis of proteins with regulated p-sites showed a strong enrichment for GO-terms related to DNA damage checkpoints (supplemental Fig. S2A; supplemental Table S3C). We note that these functional responses could not be observed at the protein expression level (supplemental Fig. S2B; supplemental Table S2A), illustrating the value of phosphoproteomics for characterizing the immediate response of cells to radiation. These data clearly show that all cell lines were able to launch a robust DDR in response to radiation.

##### In Vitro Kinase Assays Validate Novel ATM Substrates

**Fig. 4:**
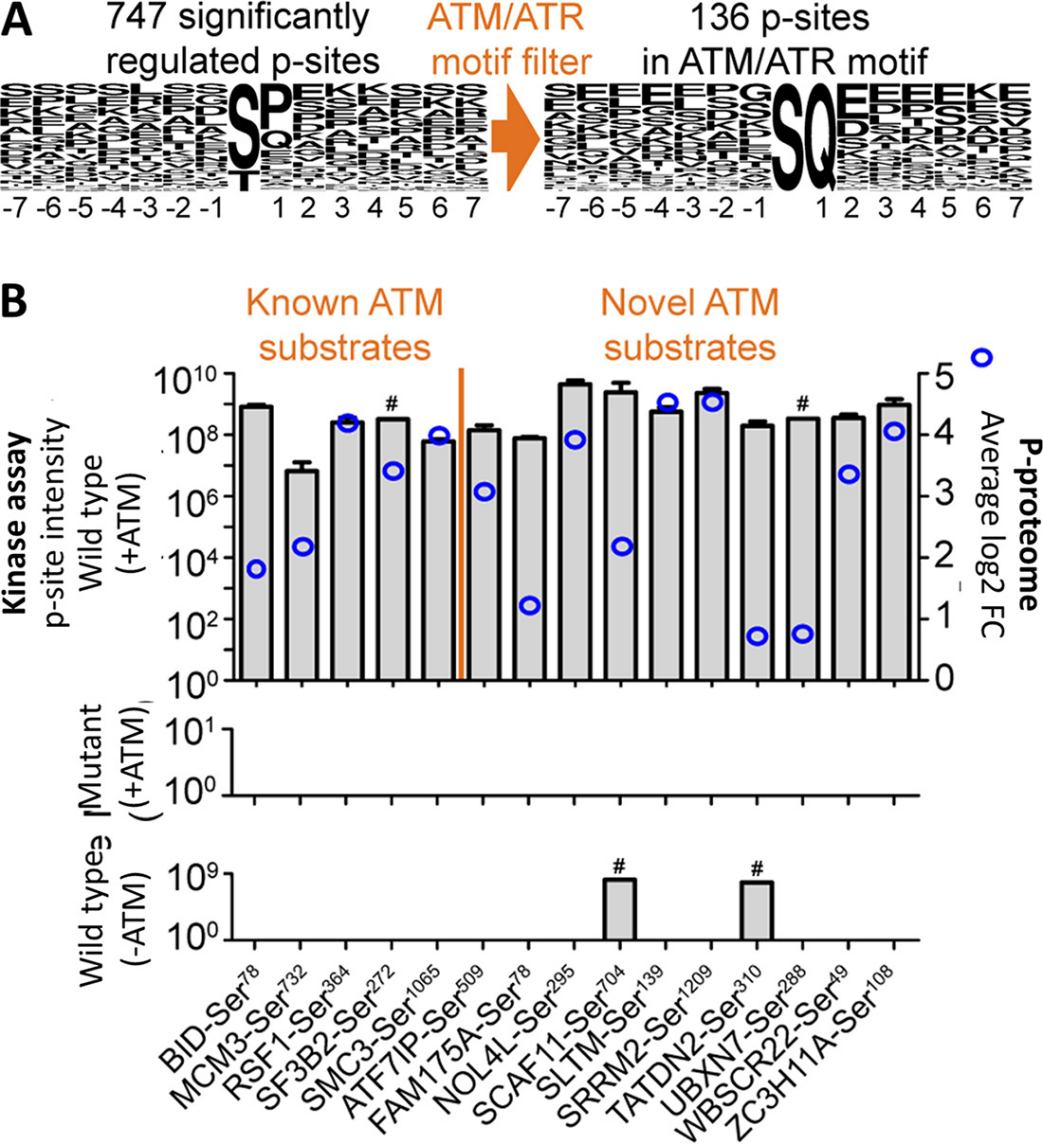
Validation of known and novel ATM kinase substrates. *A*, Sequence logo plots of radiation-mediated differentially regulated p-sites. The left plot shows all regulated p-sites, the right plot shows only those regulated p-sites that were filtered for the ATM/ATR phosphorylation motif SQ/TQ. *B*, Results of kinase assays using recombinant ATM and synthetic peptides representing the regulated SQ/TQ-containing p-sites from panel *A*. The *top panel* shows the mass spectrometric signal intensities (left *y* axis) of 15 p-peptides (separated by known and novel ATM substrates) bearing a putative ATM site after incubation with ATP and recombinant ATM. For reference, blue circles (right *y* axis) indicate the difference in p-site intensities observed in the phosphoproteomic experiment with and without irradiation (shown in Fig. 3*A*). The middle panel shows the p-site intensities of the same peptides in which the p-site was mutated to Ala. The *bottom panel* shows the p-site intensities of the WT peptides without incubation with recombinant ATM. # denotes the two p-peptides that showed some (but lower) intensity even in the absence of kinase.

Among the radiation-regulated p-sites was a striking enrichment of pSQ/pTQ sites, the consensus motif of the DDR initiating kinases ATM and ATR (39). Specifically, 136 SQ/TQ p-sites on 105 proteins were found among the 747 regulated p-sites (18%; Fig. 4*A*). The representation of SQ/TQ in the total p-proteome was only 3% (supplemental Table S4A) and almost all these SQ/TQ p-sites showed increased abundance following irradiation, consistent with the presumed activation of ATM (and ATR). Among these sites is the well-established ATM autophosphorylation site ATM-Ser^1987^ and the ATM substrate CHEK1-Ser^317^ as well as many potential novel substrates. Protein-protein interaction analysis using STRING showed that many of the regulated SQ/TQ p-sites may represent new members of an ATM signaling network with functional relevance for DDR (supplemental Fig. S3). In order to validate candidate substrates biochemically, an *in vitro* ATM assay was performed for all ATM motif-containing p-sites using synthetic peptides, recombinant ATM and LC–MS/MS for p-site identification and quantification. Motif-specificity was assessed by the inability of ATM to phosphorylate Ser/Thr to Ala-mutants of the respective peptides and this criterion was also used to designate a p-site as an ATM substrate. The experiment included five known ATM targets BID-Ser^78^ (40) Mcm3-Ser^732^ (41), RSF1-Ser^364^ (42), SF3B2-Ser^272^ (43), and SMC3-Ser^1065^ (44) and all showed specific phosphorylation by ATM (Fig. 4*B*; supplemental Table S4B–S4D). The assay also validated 10 novel ATM substrates including FAM175A-Ser^48^ (also known as ABRAXAS1-Ser^48^). ATM has previously been shown to phosphorylate FAM175A at Ser^404/406^ and the novel p-site further underlines the proteins' role in DNA damage resistance, DNA repair, and cell cycle checkpoint control (45). ATM-mediated phosphorylation of ATF7IP (MCAF1) at Ser^509^ may provide insights into how its dual function as a transcriptional activator and repressor can be modulated (46). More generally, the novel ATM substrates NOL4L-Ser^295^, SCAF11-Ser^704^, SLTM-Ser^139^, SRRM2-Ser^1209^, TATDN2-Ser^310^, UBXN7-Ser^288^, WBSCR22-Ser^49^ and ZC3H11A-Ser^108^ illustrate that the phosphoproteomic data reported here can help to functionalize these proteins, specifically in the context of radiation-induced DNA damage response. The authors note that further experiments would be necessary to show that the aforementioned p-sites are also ATM substrates in cells.

##### Radiosensitive and Resistant PDAC Cells Show Strong Abundance Differences in Apoptosis Related Proteins

**Fig. 5:**
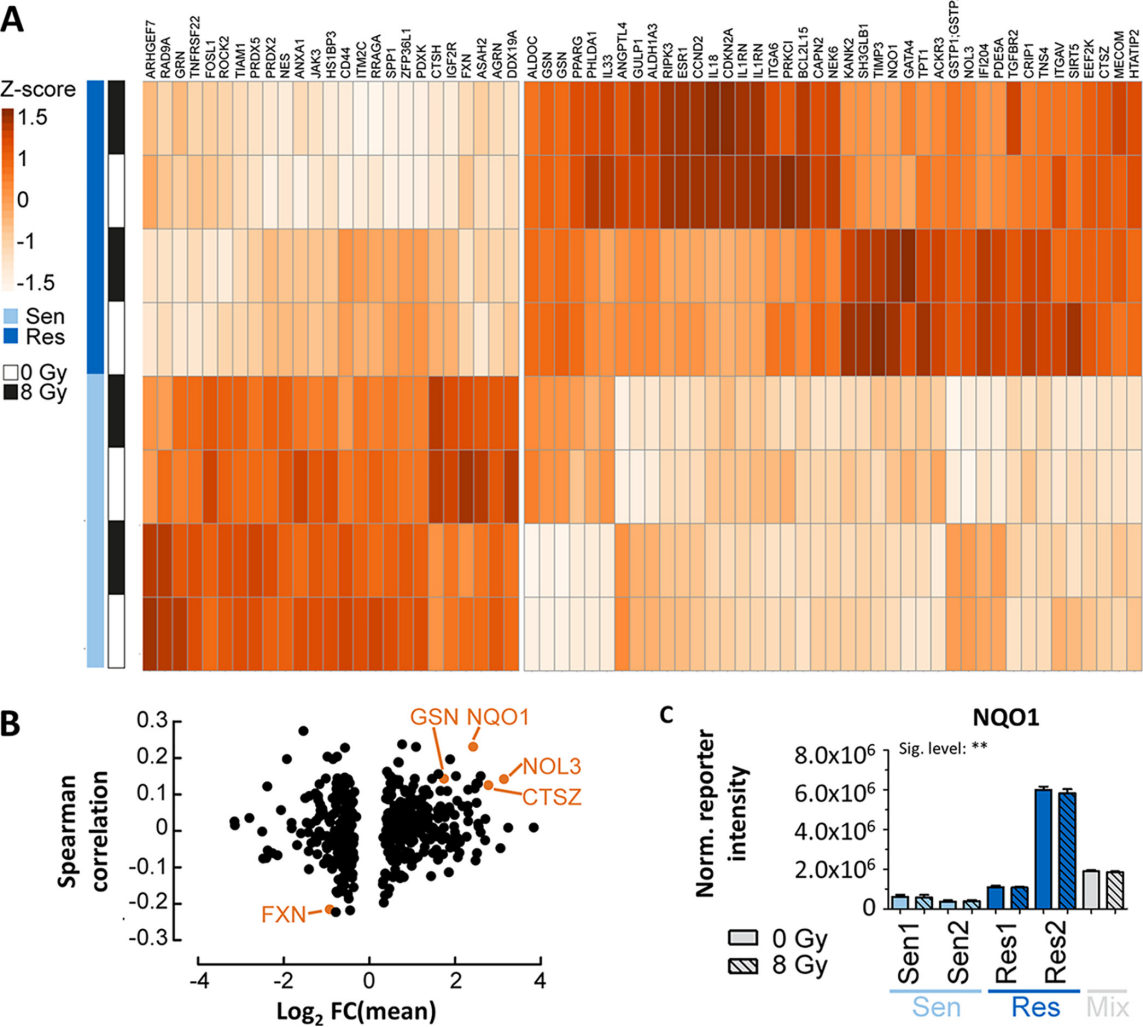
Proteome expression differences between radiosensitive and radioresistant cell lines. *A*, Heat map illustrating protein expression differences (z-scored fold changes) for apoptosis related proteins between radiosensitive and radioresistant cells. *B*, Volcano plot of the difference in protein expression between radioresistant and radiosensitive cell lines *versus* the Spearman correlation coefficient of RNA expression data of 31 PDAC lines and their response to radiation for the same proteins. Proteins of interest are highlighted in orange. *C*, Normalized TMT reporter ion intensity for the protein NQO1 showing higher NQO1 expression in radioresistant cells compared with radiosensitive cells.

The above section focused on the characterization of radiation-mediated molecular events irrespective of whether cells respond to radiation. Here, the analysis was extended to ask the question which proteins are differentially abundant between radiosensitive and resistant cell lines. The analysis showed that 491 proteins were differentially abundant between the groups of cell lines (*t* test FDR = 0.05, s0 = 0.1; supplemental Table S5A). Nonhomologous end joining (NHEJ) and homologous recombination (HR) are major elements of DSB DNA repair following irradiation of cells but only very few proteins with respective GO-terms were among the differentially expressed proteins suggesting that neither NHEJ nor HR are major contributors to radioresistance. Instead, 66 proteins involved in apoptotic processes were strongly differentially expressed between sensitive and resistant cell lines (Fig. 5*A*; highlighted in green in supplemental Table S5A) providing a hypothesis that may explain the observed differences in phenotypic response to radiation. To consolidate this list further, results from a recent study correlating RNA expression data of 533 tumor cell lines (including 31 PDAC lines) and their response to radiation (47) were integrated with the proteomic data acquired in the present study. Even though RNA and protein levels are difficult to compare directly, it is interesting to note that some proteins that showed differential abundance also (albeit relatively weakly) correlated with response to radiation (Fig. 5*B*; supplemental Table S5B). Among the proteins with the strongest effects is NQO1 (Fig. 5*C*). This protein is intimately involved in clearing reactive oxygen species (ROS) from cells, particularly upon stress stimuli (48). This may be noteworthy as it has been reported that increased ROS scavenger activity results in low ROS levels and, therefore, to radioresistance (49). One may, therefore, speculate that increased NQO1 expression in PDAC cell lines may contribute to their ability to withstand radiation-induced ROS production. Future work along these lines may include testing if NQO1 inhibitors such as the anticoagulant dicoumarol (50) that has shown growth inhibitory effects in human pancreatic cell lines (51) can sensitize PDAC cells for radiation.

##### Phosphoproteome Differences between Radiosensitive and Resistant PDAC Cells Highlight Increased Actin Dynamics and FAK Activity

**Fig. 6:**
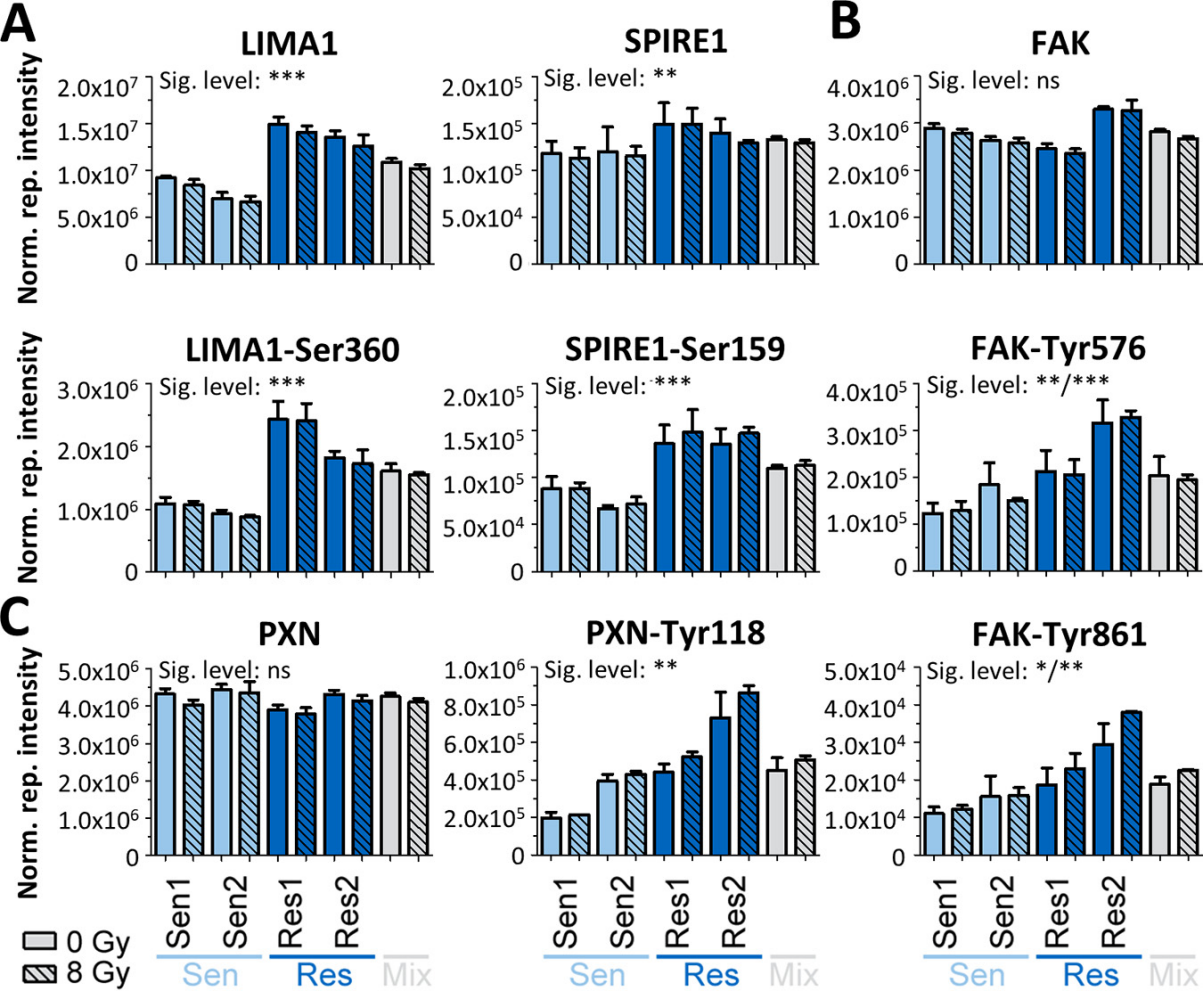
Differences in p-site intensities between radiosensitive and radioresistant cells. Normalized TMT reporter intensities of proteins and p-sites *A*, for the proteins LIMA1 and SPIRE1, *B*, for the protein FAK and *C*, for the protein PXN.

Returning to the phosphoproteomic data, abundance differences in 361 p-sites on 192 proteins were observed between radiosensitive and resistant cells (FDR = 5%, s0 = 0.1; supplemental Table S6A). GO term analysis of the underlying proteins revealed a strong enrichment for proteins with functions in cytoskeleton organization, particularly in actin dynamics (supplemental Fig. S4A; supplemental Table S5C). For example, significant differences of phosphorylation intensity were observed on Ser^360^ of the actin filament bundler LIMA1 (52) and on Ser^159^ of the actin nucleation factor SPIRE1 (53), both of which were also differentially abundant at the protein level (Fig. 6*A*; see supplemental Tables S6A for p-sites and S2A for proteins). Significantly increased phosphorylation levels but not protein levels were observed for focal adhesion kinase (FAK; Fig. 6*B*). FAK is a nonreceptor tyrosine kinase and controls several cellular processes such as cell invasion (54), epithelial-mesenchymal transition (55) and cell survival (56, 57) by kinase-dependent and kinase-independent mechanisms, further highlighting the involvement of the cytoskeleton in radioresistant cells. Elevated FAK activity in radioresistant cells was evident from an increase in phosphorylation on (1) the kinase domain activation loop Tyr^576^, which indicates formation of an activated FAK–SRC complex (58), (2) on Tyr^861^, another activating FAK-p-site (59) and (3) on paxilin (PXN)-Tyr^118^, a direct substrate of FAK (Fig. 6*C*; supplemental Fig. S4B and S4C, supplemental Table S6B). This increase in FAK activity may lead to improved cell survival in radioresistant cells and contrasts the large-scale radiation screen of cancer cells where FAK (mRNA) expression did not correlate with radiation response (47).

Consistent with the above observation that the cytoskeleton may be involved in radioresistance, migration assays showed that the mean number of migrating cells was significantly increased in the radioresistant cell line 53704PPT (*p* < 0.0001) compared with the other three cell lines (supplemental Fig. S5A and S5B; supplemental Table S6C). However, it remains to be tested if elevated FAK activity is a general characteristic of radioresistant PDAC cells or merely a feature of the cell lines tested here.

##### Kinase Inhibitors Sensitize PDAC Cells to Radiation

Motivated by the above findings, we tested if pharmacological inhibition of FAK or CHEK1 activity may lead to improved response to radiation. Defactinib is a highly potent designated FAK inhibitor that shows good selectivity assessed by results of an extensive screen of kinase inhibitor selectivity conducted by the authors (23). Similarly, Rabusertib is an exquisitely selective CHEK1 inhibitor and, therefore, phenotypic effects of this drug on cells can most likely be attributed to CHEK1 inhibition. First, the four cell lines were treated with the kinase inhibitors alone for 24 h to establish their pharmacological response. Both drugs reduced the SF in a concentration-dependent fashion compared with PBS control (supplemental Fig. S6A–S6B; supplemental Table S7A). Defactinib decreased the SF with EC50 values of 1.6 μm (53704 PPT), 1.2 μm (F5461 PPT2), 1.0 μm (5748 PPT) and 1.1 μm (53578 PPT). Rabusertib showed EC50 values of 0.34 μm (53704 PPT), 0.23 μm (F5461 PPT2), 0.14 μm (5748 PPT) and 0.26 μm (53578 PPT), respectively. No cell growth was observed for cells treated with >7.5 μm Defactinib or >500 nm Rabusertib. Therefore, Defactinib and Rabusertib concentrations were limited up to 5 μm and 375 nm respectively for further experiments. No differences in the SF of both control groups (cells treated with PBS or 0.5% DMSO) were observed. Therefore, all statistical analysis of data from the combination of kinase inhibitor and irradiation (see below) were related to the survival data of cells treated with PBS control.

**Fig. 7:**
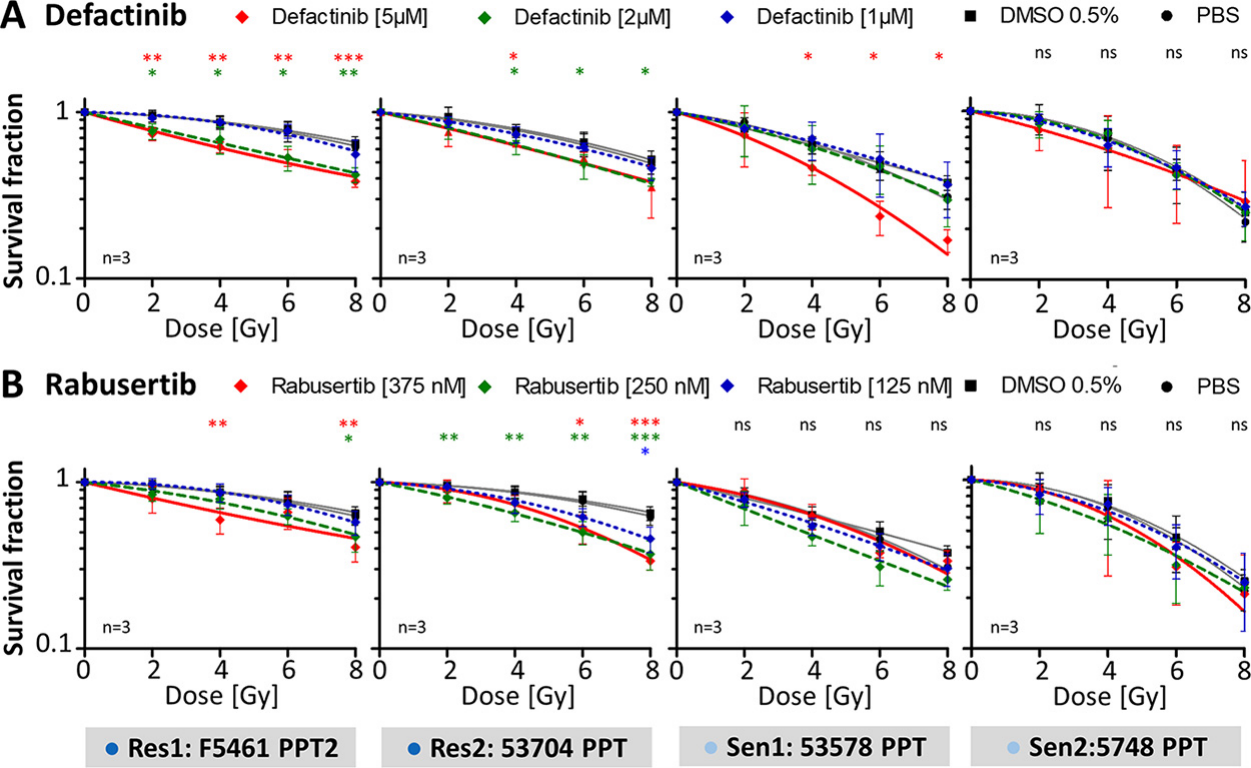
Radiosensitization of radioresistant and radiosensitive PDAC cell lines by the kinase inhibitors Defactinib and Rabusertib. Results of clonogenic survival assays of the two radioresistant cell lines (F5461PPT2 and 53704PPT) and radiosensitive cell lines (53578PPT and 5748PPT) after combined treatment irradiation with 0 Gy, 2 Gy, 4 Gy, 6 Gy and 8 Gy and *A*, Defactinib or *B*, Rabusertib. Data are normalized to the PBS treatment and are expressed as mean ± S.D. of three independent experiments (*p ≤ 0.05, **p ≤ 0.01, ***p ≤ 0.001, ****p ≤ 0.0001).

Incubating the four cell lines with Defactinib or Rabusertib for 24 h in combination with irradiation increased the efficacy of radiation (Fig. 7*A*–7*B*; supplemental Table S7B). For the radioresistant cells, the SF significantly decreased at concentrations of 2 μm Defactinib and 5 μm Defactinib (Fig. 7*A*). An even stronger radiosensitizing effect was observed for Rabusertib (Fig. 7*B*) that resulted in significantly decreased SF at 125 nm, 250 nm and 375 nm Rabusertib. The radiosensitizing effects of Defactinib and Rabusertib were confirmed by the determination of radiobiological parameters (supplemental Table S7C). For cells treated with Defactinib, the SER for D_50_ increased to 1.4 (2 μm) and 1.3 (5 μm) in 53704 PPT cells and to 1.5 (2 μm) and 1.7 (5 μm) in F5461 PPT2 cells. Combining irradiation and Rabusertib also significantly enhanced radiosensitivity with SER values of 1.3 (250 nm) and 1.3 (375 nm) for 53704 PPT cells and 1.3 (250 nm) and 1.4 (375 nm) for F5461 PPT2 cells. In contrast, neither Defactinib nor Rabusertib showed a significant radiosensitizing effect in the radiosensitive PDAC cell line 5748 PPT at any inhibitor concentration or radiation dose. The SF after combined treatment with Defactinib or Rabusertib and irradiation was also not statistically significantly reduced in 53578 PPT cells with the exception of the 5 μm Defactinib dose at 4, 6 and 8 Gy. In contrast to the radioresistant cell lines, a significantly enhanced radiosensitivity was only observed after combined treatment with irradiation and 5 μm Defactinib or 250 nm Rabusertib in both radiosensitive cell lines determined by SER > 1.2 (supplemental Table S7C).

**Fig. 8:**
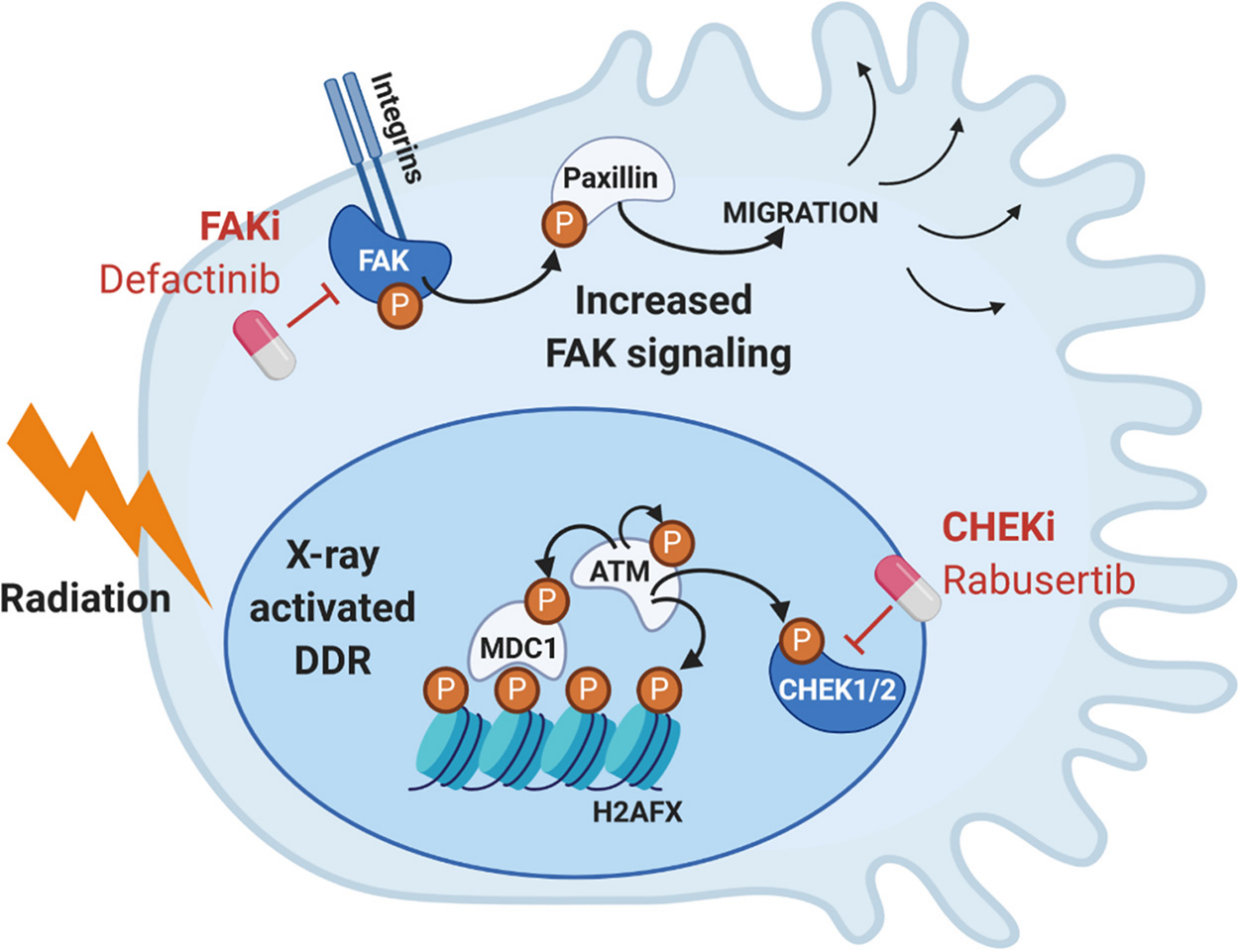
Schematic overview of the two signaling pathways, including selected p-proteins, which are activated in radioresistant cells and inhibited by Defactinib and Rabusertib leading to radiosensitization.

In summary, single treatment with kinase inhibitors Defactinib and Rabusertib resulted in a concentration-dependent decreased SF especially in radiosensitive cell lines, a statistically significant radiosensitization was mainly achieved in the radioresistant cell lines after irradiation in combination with kinase inhibition (see Fig. 8 for a schematic overview).

## DISCUSSSION

A better understanding of cancer biology in combination with the development of novel treatment modalities has generally led to improved cancer therapy and patient survival. However, the prognosis of patients with PDAC remains poor and no significant improvements in overall survival have been achieved over the past decades. The main reasons are a lack of appropriate treatment modalities and methods for detecting pancreatic cancer at an early stage resulting in a primary surgical resection in less than 20% of the cases and leaving chemotherapy and RT as major alternative treatment options (60).

Main mechanisms of ionizing radiation are the induction of DNA damage, apoptosis, changes of cell cycle distribution, autophagy, and highly reactive free radical species such as ROS leading to cell damage and cell death (61). Our data confirmed the involvement of radiation-induced DNA damage by statistically significant changes in the phosphoproteomes of several well-known DDR markers in PDAC cell lines after 8 Gy irradiation compared with unirradiated cells. Multiple factors are implicated in the development of radioresistance, including deregulated signaling pathways, oncogenic miRNA overproduction, enhanced DNA damage responses, the presence of cancer stem cells, epithelial-to-mesenchymal transition and alterations in cancer metabolism, as well as tumor microenvironment (62). Dysregulation of the mechanisms of ionizing radiation might be a major contributor both to radiation resistance and radiation sensitizing (61) and are discussed in the following.

Genetic dysregulations occurring in apoptotic signaling pathways are observed in aggressive cancer cells that limit the effects of anticancer therapies, including radiation (63). In line our own data show strong abundance differences in apoptosis related proteins in the radioresistant and radiosensitive cell lines. The tumor suppressor gene SMAD4 is mutated or deleted in 55% of PDAC and promotes pancreatic tumor progression and increases metastasis. SMAD4 depletion also induces radioresistance in pancreatic cancer both *in vitro* and *in vivo*. Mechanistically, SMAD4 depletion induces elevated levels of autophagy and ROS contributing to radioresistance, whereas autophagy and ROS inhibitors sensitized pancreatic cancer cells to radiation (9). In context of reactive free radical species, NQO1 serves as an important protective mechanism against ROS. Previous studies have demonstrated overexpression of NQO1 in various cancers including PDAC (64) and a radiation tolerance group showed an up-regulated NQO1 expression in patients with head and neck squamous cell carcinoma (65). Consistent with these findings, we demonstrated an elevated NQO1 expression in the radioresistant PDAC cell lines. Another aspect is the migratory and invasive capacity of cells, which is further increased by irradiation in some cell lines and affects radiation response (66). In line with Gray *et al.*, who demonstrated an increased invasion and migration potential of radioresistant breast cancer cell lines (67), we revealed elevated phosphorylation levels of proteins involved in cytoskeleton organization including actin dynamics and FAK activity in the radioresistant PDAC cell lines as well a strong migration phenotype in one resistant cell line.

As RT alone also often leads to unsatisfactory results because of the above discussed mechanisms of radioresistance, innovative approaches combining RT with molecularly targeted drugs may overcome the high RT resistance of PDAC. Toward this goal, we first measured the phenotypic response of a panel of 38 PDAC lines to radiation to characterize the panel. We then characterized the (phospho-) proteomes of two RT sensitive and insensitive lines in response to radiation to identify a large molecular network of known and novel DNA damage response factors centered on the kinases ATM, CHEK1 and their substrates. This analysis substantially extended the range of proteins involved in the response of cells to radiation, validated 10 new ATM substrates and suggested CHEK1 as a potential target in PDAC. In addition, the comparison of the molecular differences in the phospho-proteome between radioresistant and radiosensitive cell lines uncovered increased activity of the kinase FAK in radioresistant PDAC cells, thus offering a further way to target RT resistant PDAC.

Subsequent experiments showed that radioresistant (but not radiosensitive) PDAC cells could be sensitized by treatment with the CHEK1 inhibitor Rabusertib or the FAK inhibitor Defactinib. FAK has been implicated in regulating cancer cell migration, proliferation, cell survival and progression in PDAC (56, 57, 68). Previous studies have identified hyperactivated FAK in neoplastic PDAC cells and demonstrated enhanced tumor malignancy and correlation with poor prognosis by elevated FAK expression (68, 69). Our results of FAK inhibition using Defactinib are consistent with a described inhibition of cell proliferation and clonogenicity as well as apoptosis-induction by Defactinib in pancreatic neuroendocrine tumors (70). Single-agent FAK inhibition limited tumor progression and resulted in a doubling of survival in genetically engineered mouse models of human PDAC by inducing prolonged tumor stasis and disease stabilization (69). The observed radiosensitization is also in line with previous reports showing that siRNA silenced FAK expression radiosensitized human PDAC cell lines (71). The good selectivity of Defactinib for FAK as previously determined by Kinobead screening (23) diminishes the risk that the observed effect may be because of an off-target. Defactinib is undergoing clinical trials (phase II) including as a combination therapy with immune checkpoint inhibitors in PDAC patients (68, 69) (reviewed in (72)). This suggests that Defactinib is reasonably safe to use in humans and the results provided in this study may pave the way for testing Defactinib for targeting radioresistance in PDAC to improve the efficacy of radiation treatment in PDAC patients.

A similar approach may be envisaged for combining CHEK1 inhibition and radiation. Rabusertib has been investigated in phase II trials in combination with chemotherapy in solid tumors but the compound is not approved for use in humans and there are no current clinical trials indicating that the clinical program may have been terminated. Still, the data on Rabusertib make the point that CHEK1 inhibition may be an avenue to follow. The drug has been shown to radiosensitize HPV/p16-positive head and neck cancer cell lines (73) and other CHEK1 inhibitors showed the same in KRAS mutant rectal cancer cell lines (74). A different CHEK1 inhibitor, MK8776, has shown radiosensitizing effects in PDAC cell lines at relatively high compound concentrations (75). However, given that MK8776 is also a CDK inhibitor, it is difficult to attribute its radiosensitizing effect to the inhibition of CHEK1. In contrast, no targets other than CHEK1 have been reported for Rabusertib, which indicates that the radiosensitizing effects we observed in this study are indeed owing to the inhibition of CHEK1.

Together, the data provided in this study constitute a rich molecular resource for mechanistic studies regarding the biological response of PDAC to ionizing radiation. The study also exemplifies how phosphorylation events measured at a global scale can be used to design novel therapies. In particular, the data provide evidence to advance therapeutic concepts of FAK and CHEK1 inhibition in combination with RT in PDAC.

## DATA AVAILABILITY

The MS proteomics data and complete MaxQuant search results have been deposited with the ProteomeXchange Consortium via the PRIDE (76) partner repository and can be found using the data set identifier PXD015284. Spectra identifying post-translationally modified peptides and proteins identified on the basis of single peptide matches can be viewed in the MaxQuant Viewer included in MaxQuant software (v1.5.6.5 for the (phospho)proteomic data; v1.6.0.1 for the kinase assay data) that has also been deposited in the same location.
