# Supplementary material for: Radiosensitization by Kinase Inhibition Revealed by Phosphoproteomic Analysis of Pancreatic Cancer Cells
Source: Mol Cell Proteomics. 2020 Nov 25;19(10):1649–63. doi: 10.1074/mcp.RA120.002046 (PMC8014995; doi:10.1074/mcp.RA120.002046)
Supplement: Supplementary file 1 [file mmc1.zip › 159553_1_supp_552806_qcc38g.pdf]

Radiosensitization by kinase inhibition revealed by phosphoproteomic analysis of pancreatic cancer cells

Svenja Wiechmann[a,b,c], Elena Saupp[d], Daniela Schilling[d,e], Stephanie Heinzlmeir[a], Günter Schneider[f], Roland M. Schmid[f], Stephanie E. Combs[b,c,d,e], Bernhard Kuster[a,b,c,g], Sophie Dobiasch[b,c,d,e]

[a] *Chair of Proteomics and Bioanalytics, Technical University of Munich (TUM), 85354 Freising, Germany*
[b] *German Cancer Consortium (DKTK), Munich, Germany*
[c] *German Cancer Center (DKFZ), Heidelberg, Germany*
[d] *Department of Radiation Oncology, Technical University of Munich (TUM), Klinikum rechts der Isar, 81675 Munich, Germany*
[e] *Institute of Radiation Medicine (IRM), Department of Radiation Sciences (DRS), Helmholtz Zentrum München, 85764 Neuherberg, Germany*
[f] *Medical Clinic and Polyclinic II, Klinikum rechts der Isar, Technical University Munich, 81675 München, Germany*
[g] *Bavarian Center for Biomolecular Mass Spectrometry, Technical University of Munich (TUM), 85354 Freising, Germany*

Running title: Phosphoproteomics of radiation resistant cancer cell lines

Corresponding author:
Dr. med. Sophie Dobiasch
Sophie.Dobiasch@tum.de
Department of Radiation Oncology, Technical University of Munich (TUM)
Klinikum rechts der Isar
Ismaninger Straße 22
81675 Munich, Germany
Phone: +49 89 4140 8064
Fax: +49 89 4140 4477

Content

Supplementary Tables 2
Supplementary Table S1: Radiosensitivity of murine PDAC cell lines 2
Supplementary Table S2: Proteome and phosphoproteome dataset 2
Supplementary Table S3: Regulated phosphoproteome radiation versus control 2
Supplementary Table S4: Validation of regulated ATM substrate p-sites 3
Supplementary Table S5: Proteome expression differences resistant versus sensitive cells 3
Supplementary Table S6: Phosphoproteome expression differences resistant versus sensitive cells 3
Supplementary Table S7: Sensitization via kinase inhibition 3
Supplementary Figures 5
Supplementary Figure S1 5
Supplementary Figure S2 6
Supplementary Figure S3 7
Supplementary Figure S4 8
Supplementary Figure S5 9

Supplementary Tables

Supplementary Table S1: Radiosensitivity of murine PDAC cell lines

- **Sheet 1A:** Summary of the 38 murine PDAC cell lines including the genotype and their mean relative proliferation after irradiation with doses of 0 Gy, 2 Gy, 4 Gy, 8 Gy and 16 Gy and standard deviation (SD) using the AlamarBlue proliferation assay. Cell lines are arranged by proliferation rate at 8 Gy. (data presented in Figure 1A)
- **Sheet 1B:** Analysis of survival fraction (SF) of two representative radioresistant and two representative radiosensitive murine PDAC cell lines after irradiation with 0 Gy, 2 Gy, 4 Gy, 6 Gy and 8 Gy including the mean SF, standard deviation (SD). Statistical analysis of the SF between the different cell lines at each irradiation dose using unpaired t-test. Radiobiological parameters D_{50} (dose [Gy] to reduce survival fraction to 50%) as well as the α and β values which were derived from the linear quadratic equation $SF = \exp[-\alpha \times D - \beta \times D2]$. (data presented in Figure 1B)

Supplementary Table S2: Proteome and phosphoproteome dataset

- **Sheet 2A:** Normalized TMT reporter intensities of identified proteins in the proteome of the four PDAC cell lines upon 0 Gy or 8 Gy radiation. (data presented in Figure 2B, S1B, S2B, 5C, 6A-C)
- **Sheet 2B:** Normalized TMT reporter intensities of identified p-sites in the p-proteome of the four PDAC cell lines upon 0 Gy or 8 Gy radiation. (data presented in Figure 2B, S1B)
- **Sheet 2C:** Raw TMT reporter intensities of identified phosphopeptide-spectrum matches in the p-proteome of the four PDAC cell lines upon 0 Gy or 8 Gy radiation. (data presented in Figure 2B, S1B)
- **Sheet 2D:** Coefficients of variation (CV) of Mix 0Gy sample replicates in the phosphoproteome on p-site level and full proteome on protein level. The CV´s are reported in bins of 0.01. (data presented in Figure S1D)
- **Sheet 2E:** Pearson correlation of combined 0 Gy (Mix 0Gy) and 8 Gy (Mix 8Gy) samples in the phosphoproteome and full proteome. (data presented in Figure S1C)

Supplementary Table S3: Regulated phosphoproteome radiation versus control

- **Sheet 3A:** Significantly regulated p-sites of the four PDAC cell lines after 8 Gy radiation vs control (FDR = 1 %). (data presented in Figure 3A and B)
- **Sheet 3B:** Significantly regulated p-sites after 8 Gy radiation vs control (FDR = 1 %), which are shared or differ between sensitive and resistant cell lines. (referring to data presented in Figure 3A)
- **Sheet 3C:** GO enrichment analysis of all significantly regulated p-proteins in all four PDAC cell lines upon 8 Gy radiation vs. control. GO biological process terms are used. (data presented in Figure S2A)

Supplementary Table S4: Validation of regulated ATM substrate p-sites

- **Sheet 4A:** Significantly regulated p-sites of the four PDAC cell lines after 8 Gy radiation vs control (FDR = 1 %) bearing the ATM motif. (data presented in Figure 4A and S3)
- **Sheet 4B:** Sequences of the synthesized wildtype and mutant peptides for the in vitro ATM assay. Allocation to pool 1 and pool 2 is additionally indicated. (data presented in Figure 4B)
- **Sheet 4C:** Intensities of the p-sites of identified substrates as well of the p-sites of corresponding mutant peptides in the in vitro ATM assay. (data presented in Figure 4B)
- **Sheet 4D:** List of all PSMs and quantification values for all screened peptides in the ATM assay (based on the evidence.txt file of MaxQuant). (data presented in Figure 4B)

Supplementary Table S5: Proteome expression differences resistant versus sensitive cells

- **Sheet 5A:** Significantly regulated proteins of the two radioresistant PDAC cell lines vs the two radiosensitive PDAC cell lines (FDR = 5 %). Annotation of the proteins to the GO terms DSB repair via NHEJ (GO:0006303), DSB repair via HR (GO:0000724) and apoptotic process (GO:0006915) are indicated. (data presented in Figure 5A/C)
- **Sheet 5B:** Log2 FC values of the proteomic dataset and Spearman coefficients by Yard et al. (2016) of all significantly regulated radioresistance proteins. (data presented in Figure 5B)
- **Sheet 5C:** GO enrichment analysis of all significantly regulated p-proteins in radioresistant vs. radiosensitive cell lines. GO biological process terms are used. (data presented in Figure S4A)

Supplementary Table S6: Phosphoproteome expression differences resistant versus sensitive cells

- **Sheet 6A:** Significantly regulated p-sites of the two radioresistant PDAC cell lines vs the two radiosensitive PDAC cell lines (FDR = 5 %). Annotation of the p-proteins to the GO terms DSB repair via NHEJ (GO:0006303), DSB repair via HR (GO:0000724) and apoptotic process (GO:0006915) are indicated. (data presented in Figure 6A-C)
- **Sheet 6B:** Western blot intensity of FAK protein and FAK-Tyr576/577 levels in all four PDAC cell lines. (data presented in Supplementary Figure S4B-C)
- **Sheet 6C:** Migration behavior of cell lines determined by transwell migration assay including the mean cell number/field and standard deviation (SD) as well as statistical analysis using unpaired t-test. (data presented in supplementary Figure S5A)

Supplementary Table S7: Sensitization via kinase inhibition

- **Sheet 7A:** Analysis of SF of the two radioresistant and radiosensitive cell lines after single treatment with different concentration of the kinase inhibitors Defactinib and Rabusertib as well as determination of EC_{50} (inhibitor concentration to reduce effect on SF to 50%). (data presented in Figure S6A-B)

- **Sheet 7B:** Analysis of survival fraction (SF) of two radioresistant and two radiosensitive murine PDAC cell lines after combined treatment of kinase inhibition and irradiation with 0 Gy, 2 Gy, 4 Gy, 6 Gy and 8 Gy. Mean SF, SD, number of experiments, are shown. Statistical significance between treated groups (kinase inhibitor and or irradiation) and the untreated PBS control group was determined by multiple t-tests using the Holm-Sidak method. (data presented in Figure 7A-B)
- **Sheet 7C:** Radiobiological parameters including D_{50} (dose [Gy] to reduce survival fraction to 50%), α and β values derived from the linear quadratic equation SF = exp [−α × D −β × D2] and sensitizing enhancement ratio (SER = D_{50} (irradiation)/D_{50} (irradiation and drug)) are shown. The SER was calculated in relation to the respective control cells treated with PBS. A SER greater than 1.20 is indicative for radiosensitization (indicated in bold). (referring to data presented in Figure 7)

Supplementary Figures

Supplementary Figure S1

Accompanying main Figure 2

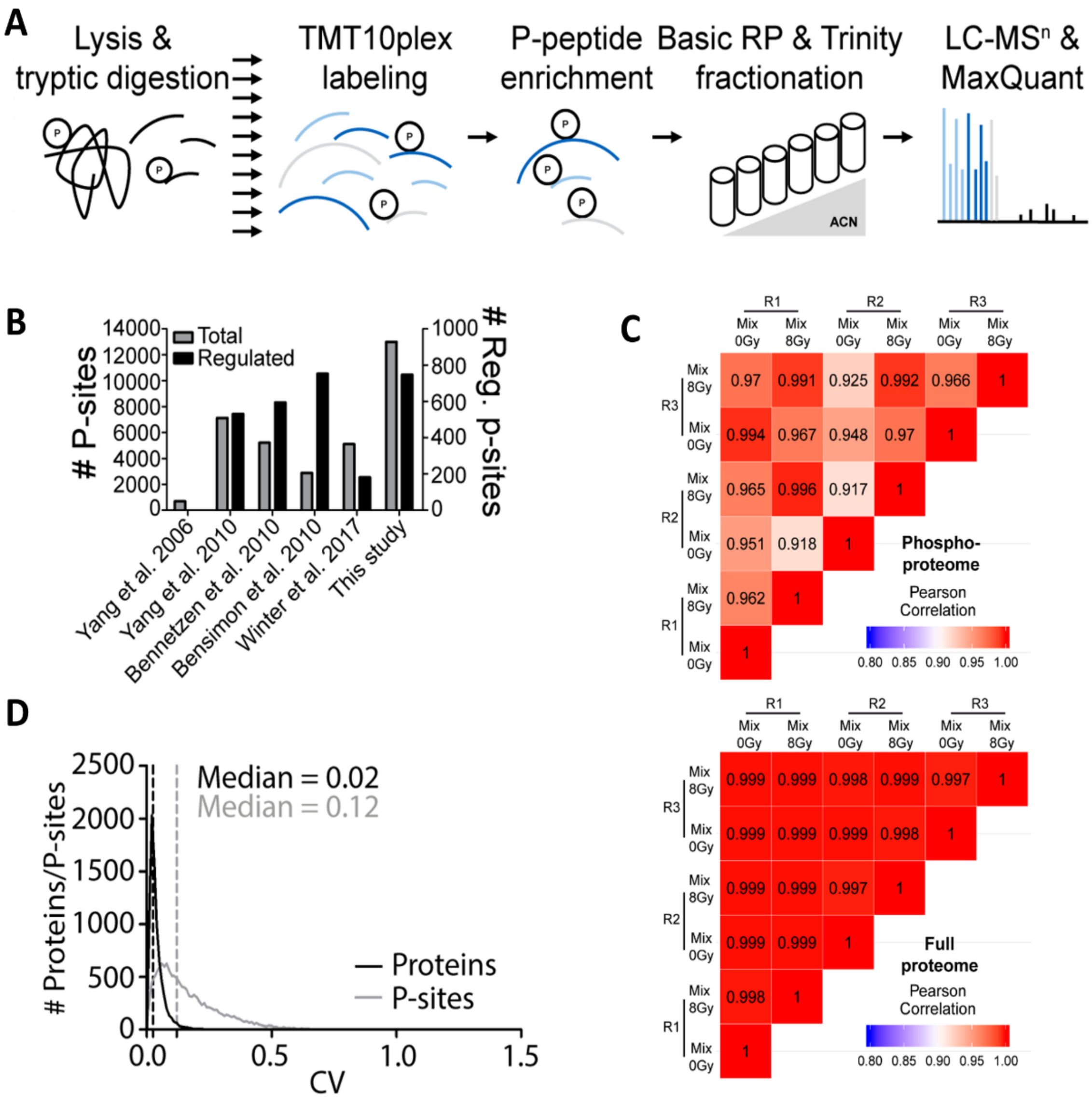

Supplementary Figure S1: Proteomic workflow and quality metrics. **A.** Schematic representation of the proteomic workflow. **B.** Comparison of the number of p-sites identified in this study and as published in the prior literature. **C.** Pearson correlation coefficients comparing replicates of the proteomic and phosphoproteomic experiments. **D.** Distribution of the coefficient of variation for proteins and p-sites between replicate proteomic and phosphoproteomic experiments.

Supplementary Figure S2

Accompanying main Figure 2 and 3

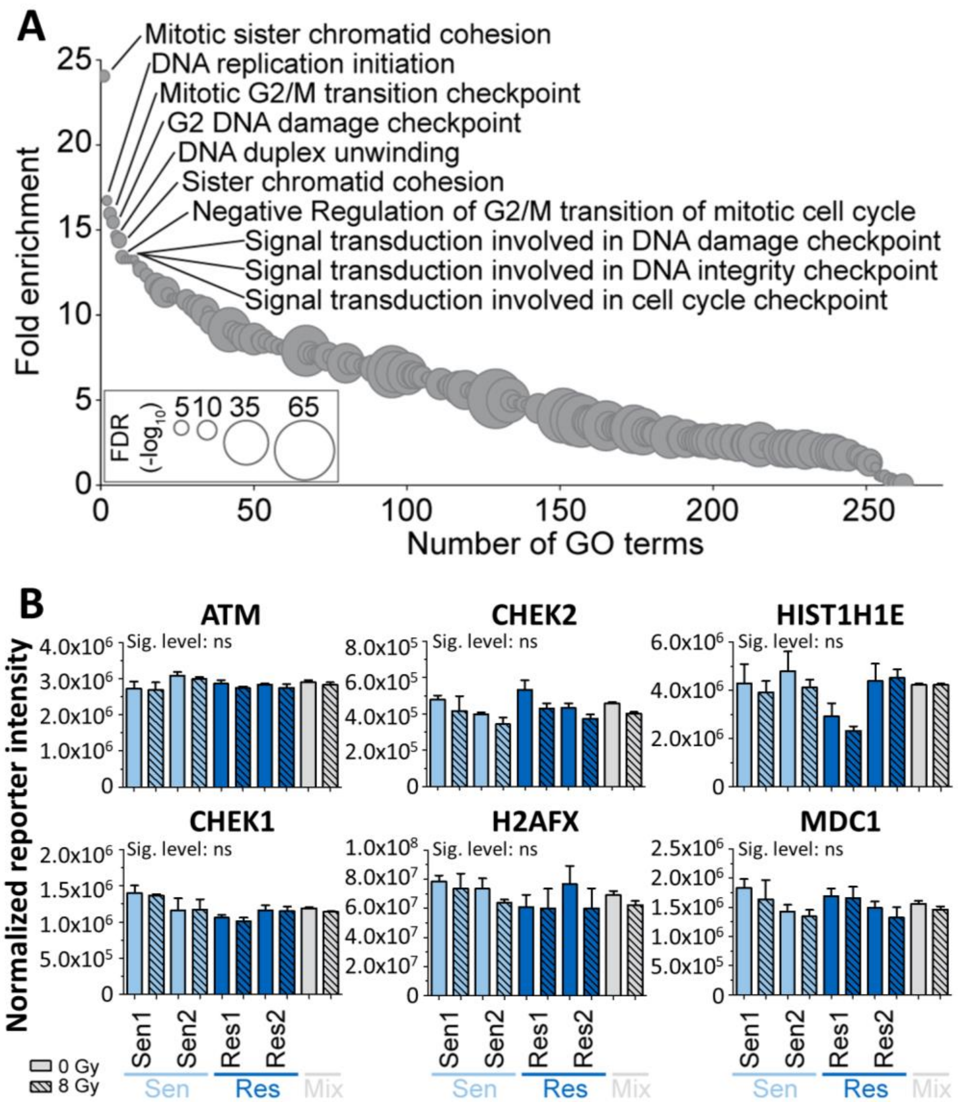

Supplementary Figure S2: A. Distribution of the number of GO-terms associated with differentially regulated p-sites comparing PDAC cells with and without radiation, the enrichment over all identified p-sites (y-axis) and the (negative log10) false discovery rate of the detected enrichment (circle size). **B.** Protein expression levels for the proteins shown in main Figure 3.

Supplementary Figure S3

Accompanying main Figure 4

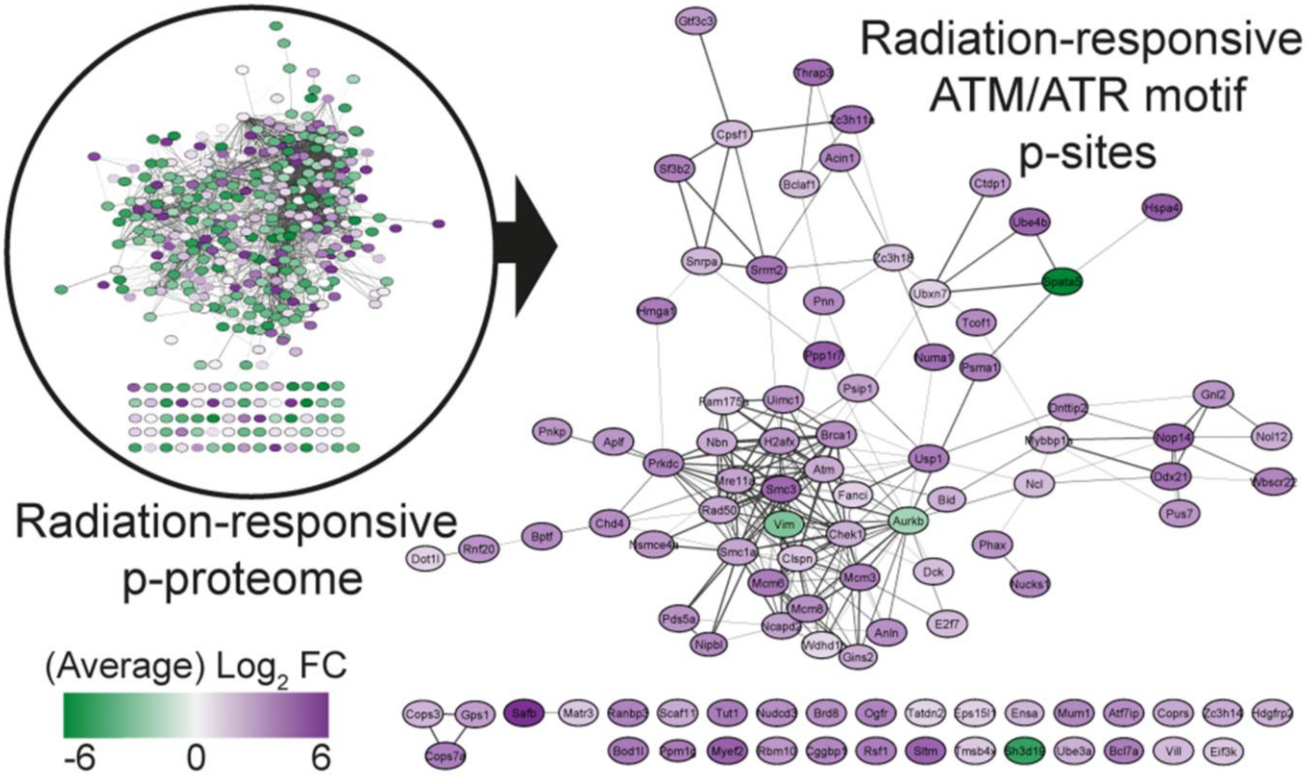

Supplementary Figure S3: Network analysis of the radioresponsive PDAC proteome. The ‘hairball’ in the black circle shows the results of a STRING analysis of all proteins with radiation regulated p-sites. The enlarged network only shows those proteins that contain regulated p-sites that harbor the ATM/ATR kinase substrate motif SQ or TQ. Nodes are colored by effect size. For simplicity, the effects of all p-sites on a given proteins were averaged. It should be noted that for the ATM/ATR network, almost all effects are positive which justifies this simplification.

Supplementary Figure S4

Accompanying main Figure 6

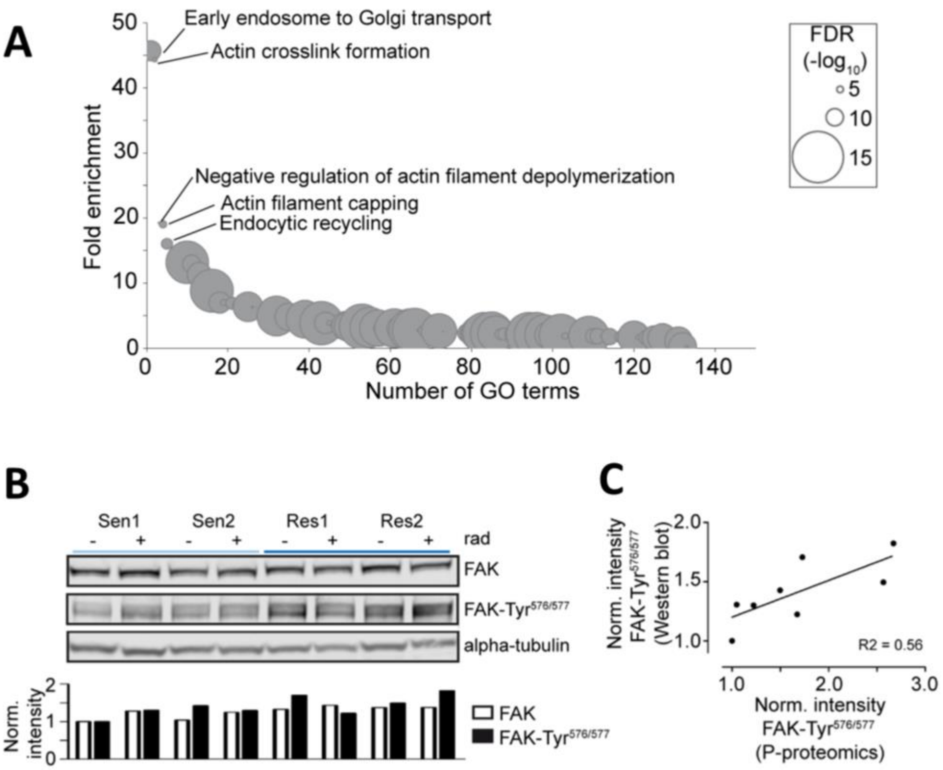

Supplementary Figure S4: A. Same as Figure S2 but for differentially regulated p-sites comparing radioresistant and radiosensitive cell lines. **B.** Western blot for total alpha-tubulin (loading control), total FAK and FAK-$Tyr^{576/577}$ (the latter indicating active FAK kinase). Quantification results of FAK and pFAK bands are placed underneath. **C.** Correlation analysis of normalized FAK-Tyr576/577 in the p-proteomic experiment and Western Blot. The R2 is displayed.

Supplementary Figure S5

Accompanying main Figure 6

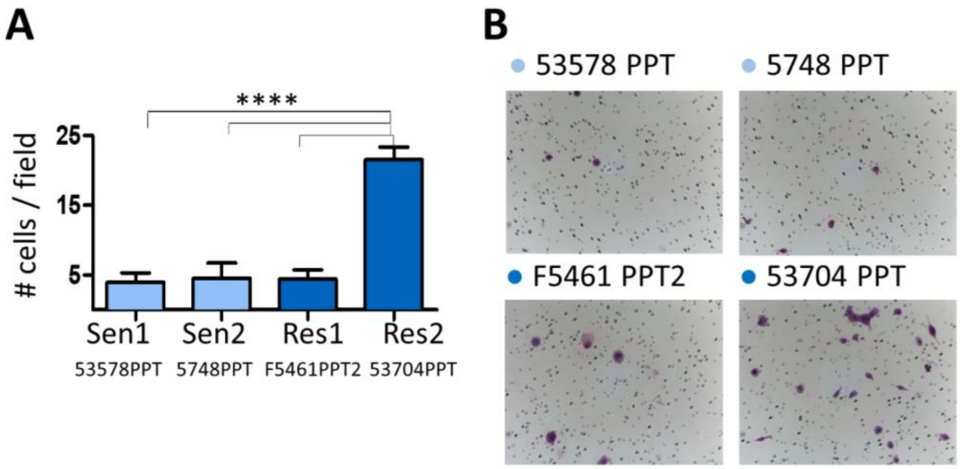

Supplementary Figure S5: A. Results of transwell migration assays for the two radiosensitive and radioresistant cell lines. Data are expressed as mean ± SD of at least 5 independent experiments (****$p \leq 0.0001$). **B.** Representative images of transwell migration assays at 10x magnification.

Supplementary Figure S6

Accompanying main Figure 7

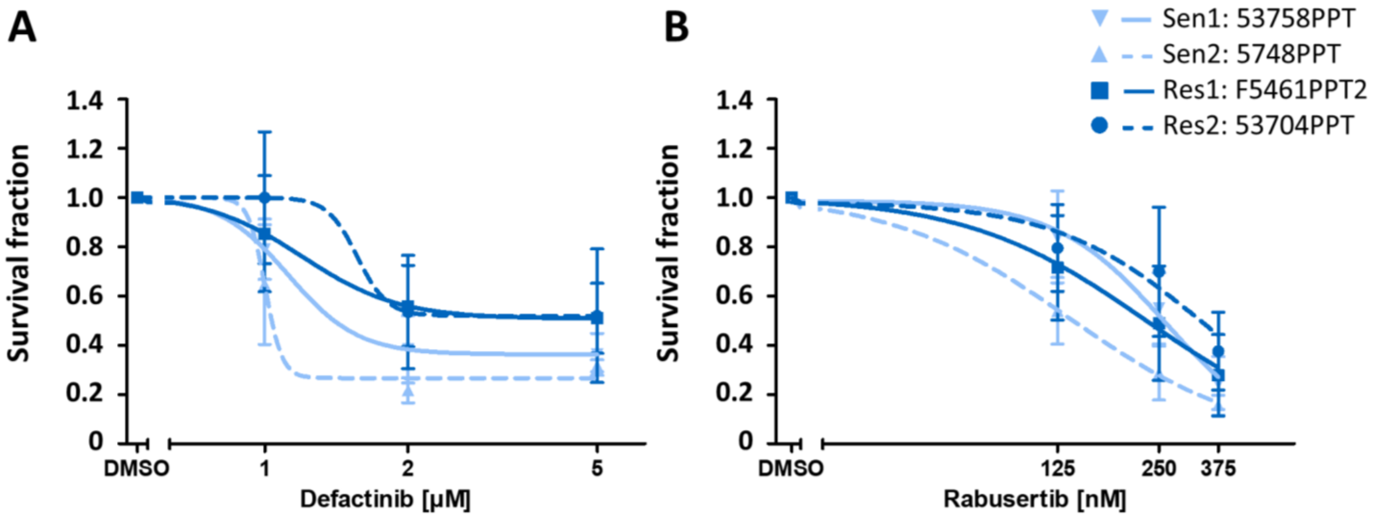

Supplementary Figure S6: Reduction of cell survival after single inhibitor treatment with Defactinib and Rabusertib. Clonogenic survival of the two radioresistant and radiosensitive cell lines after single treatment with different doses of the kinase inhibitors **A.** Defactinib and **B.** Rabusertib.
